# Aged‐senescent cells contribute to impaired heart regeneration

**DOI:** 10.1111/acel.12931

**Published:** 2019-03-10

**Authors:** Fiona C. Lewis‐McDougall, Prashant J. Ruchaya, Eva Domenjo‐Vila, Tze Shin Teoh, Larissa Prata, Beverley J. Cottle, James E. Clark, Prakash P. Punjabi, Wael Awad, Daniele Torella, Tamara Tchkonia, James L. Kirkland, Georgina M. Ellison‐Hughes

**Affiliations:** ^1^ School of Basic and Medical Biosciences, Faculty of Life Sciences & Medicine Kings College London London UK; ^2^ Robert and Arlene Kogod Center on Aging Mayo Clinic College of Medicine Rochester Minnesota; ^3^ School of Cardiovascular Medicine & Sciences, Faculty of Life Sciences & Medicine Kings College London London UK; ^4^ National Heart and Lung Institute Imperial College London London UK; ^5^ Barts Health NHS Trust London UK; ^6^ Molecular and Cellular Cardiology, Department of Medical and Surgical Sciences Magna Graecia University Catanzaro Italy

**Keywords:** aging, cardiac regeneration, cardiac repair, myocardial infarction, p16INK4a, progenitor cells, senescence, senolytics, senescence‐associated secretory phenotype

## Abstract

Aging leads to increased cellular senescence and is associated with decreased potency of tissue‐specific stem/progenitor cells. Here, we have done an extensive analysis of cardiac progenitor cells (CPCs) isolated from human subjects with cardiovascular disease, aged 32–86 years. In aged subjects (>70 years old), over half of CPCs are senescent (p16^INK4A^, SA‐β‐gal, DNA damage γH2AX, telomere length, senescence‐associated secretory phenotype [SASP]), unable to replicate, differentiate, regenerate or restore cardiac function following transplantation into the infarcted heart. SASP factors secreted by senescent CPCs renders otherwise healthy CPCs to senescence. Elimination of senescent CPCs using senolytics abrogates the SASP and its debilitative effect in vitro. Global elimination of senescent cells in aged mice (INK‐ATTAC or wild‐type mice treated with D + Q senolytics) in vivo activates resident CPCs and increased the number of small Ki67‐, EdU‐positive cardiomyocytes. Therapeutic approaches that eliminate senescent cells may alleviate cardiac deterioration with aging and restore the regenerative capacity of the heart.

## 1 INTRODUCTION

Aging is the greatest risk factor for many life‐threatening disorders, including cardiovascular diseases, neurodegenerative diseases, cancer and metabolic syndromes (St Sauver et al., 2015). Aging leads to increased cellular senescence in a number of tissues and is frequently associated with increased expression of the senescence biomarker, p16^Ink4a^ (also known as Cdkn2a), resistance to apoptosis, and impaired proliferation and tissue regeneration (Jeyapalan & Sedivy, 2008; Krishnamurthy et al., 2004; Sharpless & DePinho, 2007). Senescent cells disrupt tissue structure and function through their senescence‐associated secretory phenotype (SASP), consisting of pro‐inflammatory cytokines, chemokines, ECM‐degrading proteins and other factors, which have deleterious paracrine and systemic effects (Tchkonia, Zhu, Deursen, Campisi, & Kirkland, 2013; Xu, Palmer, et al., 2015; Xu, Tchkonia, et al., 2015). Remarkably, even a relatively low abundance of senescent cells (10%–15% in aged primates) is sufficient to cause tissue dysfunction (Herbig, Ferreira, Condel, Carey, & Sedivy, 2006; Xu et al., 2018).

To test whether senescent cells are causally implicated in age‐related dysfunction and whether their removal is beneficial, J. L. Kirkland, T. Tchkonia, J. van Deursen (Mayo), D. Baker (Mayo) and colleagues made use of a promoter for the biomarker for senescence, p16^INK4a^, and an inducible “suicide” gene designed by P. Scherer *et al*. (Pajvani et al., 2005) to develop a novel transgene, INK‐ATTAC, to permit inducible elimination of p16^INK4a^‐positive senescent cells upon administration of a drug (AP20187) (Baker et al., 2011). In these mice, eliminating a relatively small proportion (~30%) of senescent cells extends health span and prevents the development of multiple age‐related morbidities in both progeroid and normal, chronologically aged mice (Baker et al., 2011; Farr et al., 2017; Ogrodnik et al., 2017; Roos et al., 2016; Schafer et al., 2017; Xu et al., 2018). Moreover, late‐life clearance attenuated the progression of already established age‐related disorders (Xu et al., 2018). To be applicable to humans, Kirkland and collaborators have identified a new class of drugs named senolytics in work that paralleled, but was not dependent on development of INK‐ATTAC mice. Through exploiting senescent cells’ dependence on specific prosurvival pathways, senolytics specifically kill senescent cells without affecting proliferating or quiescent, differentiated cells (Kirkland & Tchkonia, 2015, 2017; Kirkland, Tchkonia, Zhu, Niedernhofer, & Robbins, 2017). Recent studies have documented senolytic drugs for the selective clearance of senescent cells from “aged” tissues, which led to delayed acquisition of age‐related pathologies (Tchkonia & Kirkland, 2018). Recently, the Kirkland lab has demonstrated that transplanting relatively small numbers of senescent preadipocyte cells into young (6 month old) mice causes persistent physical dysfunction, measured through maximal speed, hanging endurance and grip strength, 1 month after transplantation. Transplanting even fewer senescent cells into older (17 month old) recipients had the same effect and reduced survival, indicating the potency of senescent cells in shortening health‐ and lifespan. Intermittent oral administration of the senolytics, dasatinib (D), a FDA‐approved tyrosine kinase inhibitor, and quercetin (Q), a flavonoid present in many fruits and vegetables, to senescent cell‐transplanted young mice and naturally aged mice alleviated physical dysfunction and increased post‐treatment survival by 36% while reducing mortality hazard to 65% (Xu et al., 2018). Altogether these data indicate that cellular senescence is causally implicated in generating age‐related phenotypes and that systemic removal of senescent cells can prevent or delay tissue dysfunction, physical dysfunction and extend health‐ and lifespan.

Like other organs, the adult mammalian heart has the capacity, albeit low, to self‐renew cardiomyocytes over the human lifespan (Bergmann et al., 2009), and genetic fate mapping models show that one source of new cardiomyocytes is a population of resident stem cells (Ellison et al., 2013; Hsieh et al., 2007). Multiple labs have shown the mammalian heart, including human, to harbour self‐renewing, clonogenic, multipotent cardiac stem and progenitor cells (CSCs or CPCs; abbreviated hereafter as CPCs) with regenerative potential in vivo (Ellison‐Hughes & Lewis, 2017; Vicinanza et al., 2017). An undisputed mechanism of action of CPC transplantation into the infarcted myocardium is their secretome reparative potential, which through acting in a paracrine manner improves cardiomyocyte survival, limits fibrosis, induces angiogenesis, new cardiomyocyte formation and restores cardiac function (Broughton et al., 2018).

Our lab has defined the CPC population to be Sca‐1^pos^/c‐kit^pos^/CD31^neg^/CD45^neg^/Tryptase^neg^ (Ellison et al., 2013; Vicinanza et al., 2017) distinguishing them from cardiac c‐kit^pos ^endothelial (CD31^pos^) and mast (CD45^pos^/Tryptase^pos^) cells. The CPCs are a rare population in the adult heart, with ~1% evidencing cardiomyogenic regeneration potential (Vicinanza et al., 2017). Recently, considerable confusion has emanated over the significant cardiomyogenic potential of CPCs purported using Kitcre knock‐in or dual recombinases‐mediated cell tracking mouse models (Li et al., 2018; van Berlo et al., 2014). However, these models do not tag or specifically lineage trace the CPCs, neither have the investigators isolated, characterized or transplanted CPCs (according to the Sca‐1^pos^/c‐kit^pos^/CD31^neg^/CD45^neg^/Tryptase^neg^ phenotype given above) from these mice to test their stem cell and regenerative properties. Moreover, the c‐kitCre null allele produced by Cre insertion fails to recombine the CPCs in the Kitcre mice and there is a severe defect in CPC myogenesis produced by the c‐kit hemizygosity (Vicinanza et al., 2018).

Cardiac aging and pathology affects the activity and potency of CPCs (Castaldi et al., 2017; Cesselli et al., 2011; Torella et al., 2004), which translates into a diminished capacity of the aged and diseased myocardium to maintain homoeostasis, and repair and regenerate following injury (Ellison & Lewis 2017). The aging *milieu* might therefore limit the success of cell transplantation therapies where the outcome is direct cardiogenic differentiation of transplanted cells and/or stimulation of endogenous regenerative mechanisms. As the majority of cardiovascular disease patients in need of regenerative therapies are of advanced age, regulation of CPC and cardiovascular aging/senescence is mission critical.

Here, we have carried out an extensive analysis of CPCs in the human failing heart with advanced age and showed the accumulation of senescent‐CPCs, which exhibit diminished self‐renewal, differentiation and regenerative potential in vivo. We show that Senescent‐CPCs have a SASP that negatively affects healthy non‐senescent, cycling‐competent CPCs, rendering them senescent. Clearing the senescent‐CPCs using senolytics attenuates the SASP and its effect on promoting senescence in vitro. The effects of global elimination of senescent cells on the heart and its regenerative capacity have not been elucidated. We report novel data that show systemic elimination of senescent cells in vivo in aged mice using senolytics (D + Q) or using the “suicide” transgene, INK‐ATTAC with administration of AP20187, results in CPC activation and increased number of small, immature, Ki67+ or EdU+ cardiomyocytes in the aged mouse heart.

## 2 RESULTS

### 2.1 CPCs exhibit a senescent phenotype with increased age

Human CPCs were isolated from biopsies of right atria, obtained from subjects who had given informed consent before undergoing cardiac surgery (aortic disease, valve disease, coronary artery bypass graft (CABG) or multiple diseases), using sequential enzymatic digestion and dissociation, Optiprep density gradient to remove large debris, followed by magnetic activated cell sorting (MACS) (Supporting Information Figure S1a). CPCs were magnetically enriched based upon a CD45‐negative, CD31‐negative, CD34‐negative and c‐kit‐positive sorting strategy (Smith et al., 2014; Vicinanza et al., 2017) (Supporting Information Figure S1b). Despite being recognized as a CPC marker, cells were not sorted for Sca‐1 because its homology has not been confirmed in any species other than mouse. By flow cytometry analysis, CPCs showed expression of other recognized CPC markers, such as CD90 (37 ± 0.4%), CD166 (41 ± 1%), CD105 (13 ± 1%) and CD140α (5 ± 0.4%) (Supporting Information Figure S1c). There were no differences in the number of CPCs isolated from old (>70 years) subjects, compared to subjects <70 years. We also found no differences in the number of CPCs isolated from male or female, or from those subjects with valve disease, coronary disease or aortic disease (Supporting Information Figure S1d).

**Figure 1:**
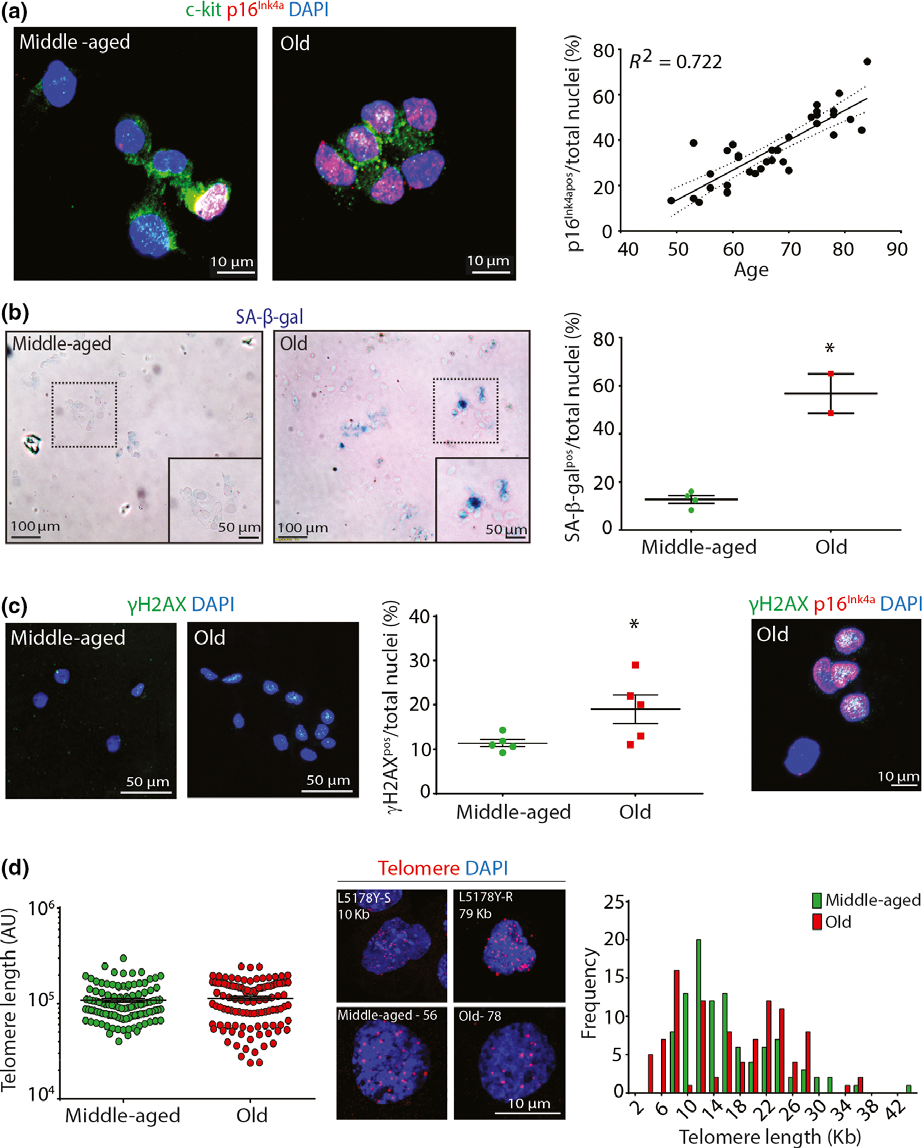
Over half of cardiac progenitor cells (CPCs) in the aged human heart are senescent (a) Representative immunofluorescence images and quantification of c‐kit^pos^ p16^INK4Apos ^CPCs, (*n* = 35 donors), (b) c‐kit^pos^ SA‐β‐gal^pos^ CPCs (*p = 0.0014; *n* = 2–4), (c) c‐kit^pos^ γ‐H2AX^pos^ p16^INK4A^‐expressing CPCs (*p = 0.0264; *n* = 5 donors). (d) Q‐FISH telomere length of single c‐kit^pos^ CPCs (*n* = 100 cells per group, 20 cells per donor). Representative immunofluorescence images of telomere staining, L5178Y‐S (10 kb) and L5178Y‐R (79 kb) are mouse cell lines with known telomere length. Frequency distribution histogram of CPC telomere length (*n* = 5 donors/group). Nuclei stained in blue by DAPI. All data are mean ± *SEM*

We isolated CPCs from 35 subjects of different genders, ages and pathologies and found a linear increase (*R* ^2^ = 0.722) in the number of freshly isolated CPCs that expressed the senescence‐associated marker, p16^INK4A^, with age (Figure 1a). No differences were evident between males and females, smokers (including ex‐smokers) and nonsmokers, diabetics and nondiabetics, and hypertensive and nonhypertensive subjects for p16^INK4A^ expression (Supporting Information Figure S2a–d), and even though a trend was apparent, we found no differences between aortic disease, valvular disease, coronary artery disease and multiple other diseases for p16^INK4A^ expression (Supporting Information Figure S2e). On average, 22 ± 9%, 31 ± 4%, 48 ± 9% and 56 ± 16% of freshly isolated CPCs expressed p16^INK4A^ isolated from 50–59, 60–69, 70–79 and 80–89 year old subjects, respectively (Figure 1a). We also found an increase (*p* < 0.05) in the number of senescence‐associated β‐galactosidase‐ (SA‐β‐gal; ~60%) and DNA damage marker, γH2AX‐positive CPCs (~20%) freshly isolated from old (71–79 years), compared to middle‐aged (54–63 years) subjects (Figure 1b,c). Moreover, p16^INK4A^‐positive CPCs co‐expressed γH2AX (Figure 1c). Further interrogation by Q‐FISH revealed that, while the average telomere length of CPCs freshly isolated from old and middle‐aged subjects’ hearts were comparable, CPCs freshly isolated from old (78–84 years) subjects’ hearts contained a 12% subpopulation with telomere length of <6 kb, which is regarded as being critically short (Figure 1d) (Canela, Vera, Klatt, & Blasco, 2007)*. *Approximately 2% of the CPCs freshly isolated from human hearts were Ki67‐positive, reflective of their mainly dormant, quiescent phenotype (Ellison et al., 2013)*. *There were no differences between middle‐aged and old subjects in number of Ki67‐positive CPCs, and we did not see any Ki67‐positive CPCs that were p16^INK4A^‐positive (Supporting Information Figure S2f). These findings indicate that the aged human heart contains an increased proportion of aged senescent‐CPCs, which could translate to their dysfunctionality.

### 2.2 CPCs from old subjects show impaired cell growth and differentiation

CPCs were isolated from five old (76–86 years) and eight middle‐aged (32–66 years) subjects, plated in growth medium, and propagated, where possible, to passage 11. CPCs isolated from two of the middle‐aged (32 and 61 years) and two of the oldest (78 and 80 years) subjects failed to grow and become established in vitro*. *Of the CPC cultures that did grow from all age groups (*n* = 9), the CPCs, from P3 to P11, gradually lost their p16^INK4A^–positive subpopulation (Supporting Information Figure S2g), likely due to the cell culture activated, cycling‐competent CPCs outgrowing their p16^INK4A^–positive senescent counterparts. CPCs maintained the phenotype of c‐kit‐positive, CD31‐negative over culture passage (Supporting Information Figure S2h). To ensure that the effect of donor age could be effectively evaluated, all in vitro cell dynamic assays were performed between P2‐P4.

**Figure 2:**
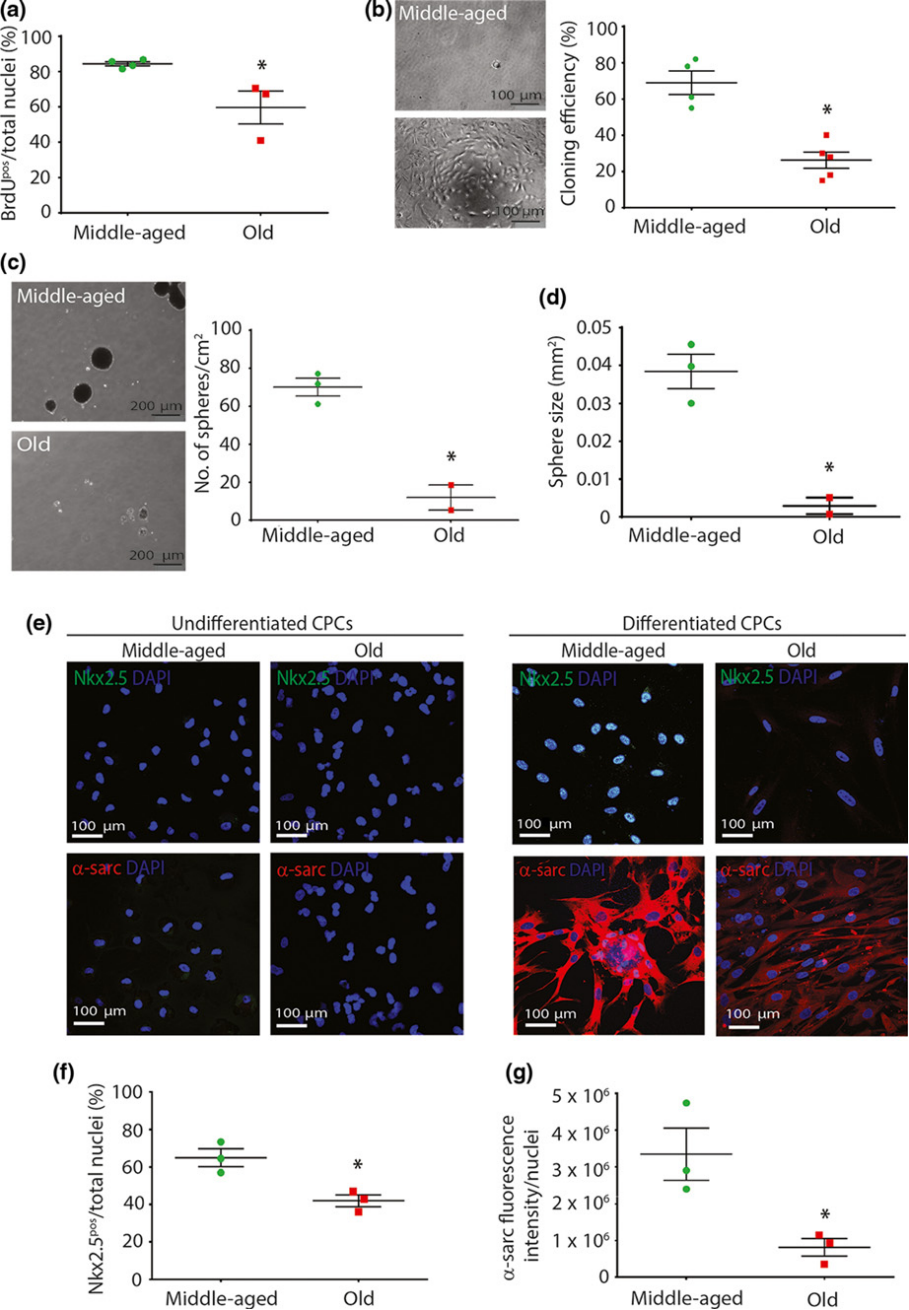
Cardiac progenitor cells (CPCs) isolated from aged hearts exhibit diminished proliferation, clonogenicity and cardiomyocyte differentiation potential (a) Quantification of CPC proliferation (*p = 0.0266; *n* = 3–4 donors), (b) single CPC‐derived clonal efficiency (*p = 0.0008, *n* = 4–5 donors), (c) CPC spherogenesis number (*p = 0.0051) and (d) size (*p = 0.01), (*n* = 2–3 donors). (e) Representative immunofluorescence images of undifferentiated and differentiated CPCs from old and middle‐aged donors. Nuclei stained in blue by DAPI. (f) Quantification of Nkx2.5^pos^ (*p = 0.0078; *n* = 3 donors) and (g) α‐sarcomeric actin expression (*p = 0.0140; *n* = 3 donors). All data are mean ± *SEM*

CPCs isolated from old (77–86 years) subjects showed decreased (*p* < 0.05) proliferation compared to CPCs isolated from middle‐aged (34–62 years) subjects (Figure 2a). CPCs deposited as a single cell in a 96‐well plate generated a greater number (*p* < 0.05) of clones if the single CPCs originated from middle‐aged (34–62 years) subjects, compared to old (76–86 years) subjects (Figure 2b). Likewise, CPCs deposited at low dilution in bacteriological dishes for the generation of spheres in suspension were greater in number and size (*p* < 0.05) for middle‐aged (34–51 years) subjects’ CPCs, compared to old (76–77 years) subjects’ CPCs (Figure 2c,d). When CPCs were plated in cardiomyocyte differentiation medium, they primed towards a cardiomyocyte‐like precursor cell type that was Nkx2.5‐positive, sarcomeric actin‐positive and middle‐aged (47–62 years) subject's CPCs had increased (*p* < 0.05) differentiation potential, compared to CPCs from old (76–77 years) subjects (Figure 2e–g). Older subject's differentiated CPC‐derived precursor cells showed disorganized sarcomeric structure (Figure 2e) and decreased (*p* < 0.05) expression (Figure 2g), compared to differentiated CPCs from younger, middle‐aged subjects.

Even though CPCs isolated from old hearts showed decreased proliferation, clonogenicity and differentiation potential, only ~50% of CPCs are senescent in old myocardium (Figure 1a); therefore, these data imply that a functionally cycling‐competent CPC population still exists in old myocardium. Indeed, single CPC‐derived clones from younger (22–33 years) and old (74–83 years) subjects were indistinguishable in terms of morphology, senescence, multipotency, self‐renewing transcript profile and differentiation (Supporting Information Figure S3). These findings suggest that CPCs age and become senescent in a stochastic, nonautonomous manner. This resembles what was seen in rat preadipocytes (Kirkland, Hollenberg, & Gillon, 1990).

### 2.3 Aged‐senescent CPCs lose their regenerative capacity in vivo

To purify for a senescent population of CPCs, we utilized the C_12_‐5‐Dodecanoylaminofluorescein Di‐β‐d‐Galactopyranoside (C12FDG) probe and sorted SA‐β‐gal‐positive CPCs through FACS (Supporting Information Figure S4a–d). We also induced senescence in CPCs pharmacologically using Doxorubicin and Rosiglitazone (Supporting Information Figure S4e–g), which we have used previously to render cells to senescence in vitro (Xu, Palmer, et al., 2015; Xu, Tchkonia, et al., 2015; Xu et al., 2018). Senescent‐CPCs, whether Doxorubicin‐, or Rosiglitazone‐induced and purified using the C12FDG probe, exhibited a senescent phenotype being p16^INK4A^–positive, Ki67‐negative and with shorter telomeres (Supporting Information Figure S5). Senescent SA‐β‐gal‐positive CPCs were nonproliferative and did not form clonal colonies when deposited as single cells, or generate spheres in vitro, compared to SA‐β‐gal‐negative, Ki67‐positive cycling‐competent CPCs (Supporting Information Figure S5). FACS phenotyping revealed decreased surface expression of the progenitor markers, c‐kit, CD90, CD105 and CD166 and increased expression of CD34 in SA‐β‐gal‐positive, senescent‐CPCs compared to SA‐β‐gal‐negative, cycling‐competent CPCs (Supporting Information Figure S6).

**Figure 3:**
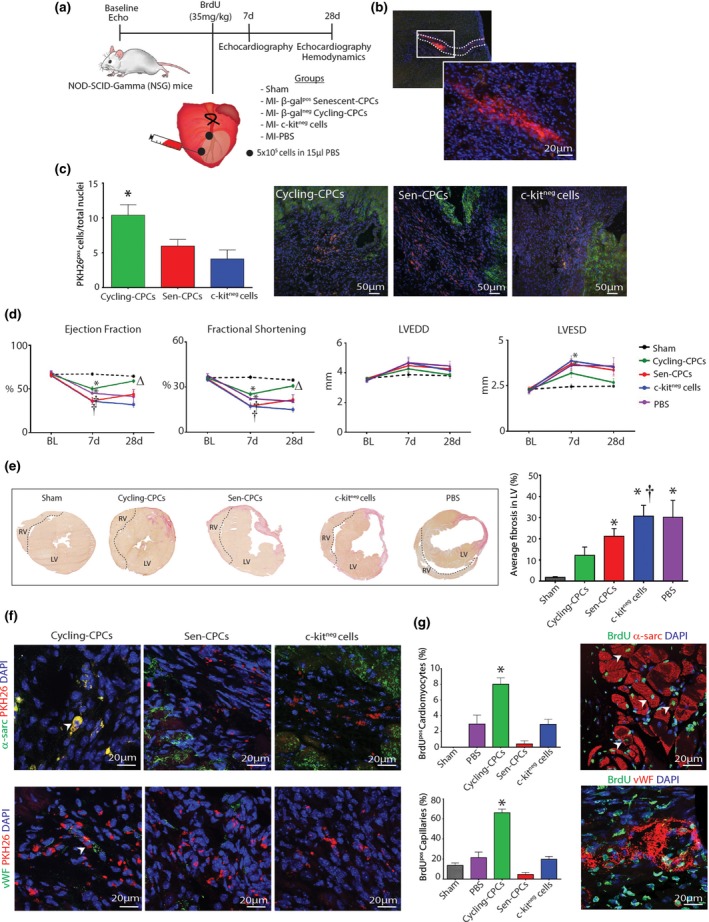
Aged‐senescent CPCs show decreased reparative potential (a) In vivo MI experimental design. (b) Representative confocal images of PKH26^pos ^CPCs engrafted in the myocardium 4 days post‐MI. (c) Quantification and representative confocal images of engraftment of PKH26^pos ^cells 28 days post‐MI (*p = 0.015 vs. c‐kit^neg ^cells, *n* = 4–5 mice). (d) Echocardiography measurements of LV ejection fraction (EF), fractional shortening (FS), left ventricular end‐diastolic diameter (LVEDD) and left ventricular end‐systolic diameter (LVESD) at baseline (BL) before MI, 7 and 28 days after MI and cell injection (*p < 0.05 vs. Sham, †*p* < 0.05 vs. Cycling‐CPCs, Δ*p *< 0.05 vs. dox‐induced Sen‐CPCs, *n* = 5–7 mice). (e) Representative micrographs and quantification of average LV fibrosis (*p < 0.05 vs. Sham; †*p* = 0.0112 vs. Cycling‐CPCs; *n* = 5–6 mice). (f) Representative confocal images of PKH26 co‐expression with α‐sarcomeric actinin and vWF (arrowheads) 28 days post‐MI. (g) Quantification and representative confocal images of BrdU^pos^/α‐sarcomeric actinin^pos^ cardiomyocytes and BrdU^pos^/vWF^pos^ capillaries (*p < 0.05 vs. all; *n* = 5–7 mice). Nuclei stained in blue by DAPI. All data are mean ± *SEM*

To determine whether the dysfunctional stem/progenitor cell properties of the senescent‐CPCs translated in vivo, we tested the regenerative capacity of doxorubicin‐induced senescent SA‐β‐gal‐positive CPCs and cycling‐competent SA‐β‐gal‐negative CPCs in the myocardial infarction‐regeneration mouse model (Figure 3a). To separate the impact of senescence from other potentially confounding aging processes operative in older mice, such as NAD deficiency, accumulation of advanced glycation end products, or accumulation of misfolded proteins, we transplanted senescent or cycling‐competent CPCs into young mice. Male, immunodeficient Nod‐SCID‐Gamma (NSG) mice were subjected to permanent ligation of the left anterior descending (LAD) coronary artery. Immediately after ligation, 5 × 10^5^ SA‐β‐gal‐positive senescent or SA‐β‐gal‐negative cycling‐competent CPCs were injected intramyocardially in 15µl of PBS at 2 sites in the border zone. To serve as a cell control, a separate set of MI‐mice were injected with 5 × 10^5^ non‐CPCs (c‐kit^neg^ cardiac‐derived cells; containing 86 ± 5% cardiac fibroblasts, 13 ± 3% vascular smooth muscle, 1 ± 1% endothelial cells (Ellison et al., 2013)). To serve as a control, a separate set of MI‐mice were injected with PBS. Sham animals were treated the same way, except ligation of LAD coronary artery was not performed and they did not receive cells but were injected with the same volume of PBS. Mice were administered BrdU via osmotic mini pumps for 14 days after MI and cell injection to track new cell DNA formation (Figure 3a). All cell populations were labelled prior to injection with PKH26 lipophilic membrane dye, which exhibited high labelling efficiency and label dye retention over population doublings in cycling‐competent CPCs and c‐kit^neg^ cardiac‐derived cells in vitro (Supporting Information Figure S7). We sacrificed a subset of MI‐mice that had been injected with 5 × 10^5^ SA‐β‐gal‐negative cycling‐competent CPCs at 4 days. There was high engraftment and survival of CPCs within the infarct/border zone at 4 days (Figure 3b) and by 28 days the engraftment was still ~10% of cycling‐competent CPCs per total nuclei in the infarct/border zone (Figure 3c). The engraftment and survival of senescent‐CPCs and c‐kit^neg^ cells at 28 days was significantly (*p* < 0.05) less (Figure 3c).

At 1 week after LAD ligation, all groups had decreased (*p* < 0.05) LV function, compared to baseline and sham controls, however, the group that had been injected with cycling‐competent CPCs had less of a decrease in LV function at 1 week, compared to the senescent‐CPC and non‐CPC c‐kit^neg^ cell groups (Figure 3d). The degree of MI represented as the % Average Area at Risk (AAR) through Evans Blue staining immediately after MI was 33.0 ± 1.6% (*n* = 5), demonstrating the operator as being extremely consistent in inducing a similar size MI injury to each mouse. At 4 weeks after LAD ligation, MI hearts that had received cycling‐competent CPCs showed an improvement (*p* < 0.05) in LV ejection fraction (EF), fractional shortening (FS), left ventricular end diastolic diameter (LVEDD) and left ventricular end systolic diameter (LVESD), which had almost returned to baseline values and to that of Sham controls (Figure 3d).

This extent of LV improvement was not apparent in MI‐mice that were injected with SA‐β‐gal‐positive senescent‐CPCs, PBS or the non‐CPC c‐kit^neg^ cell group that showed no recovery with worsened LV function and were all in heart failure at 4 weeks (Figure 3d). To accompany these functional changes, cycling‐competent CPC injection resulted in a decreased (*p* < 0.05) infarct size, whereas SA‐β‐gal‐positive senescent‐CPCs, PBS or non‐CPC c‐kit^neg^ cells did not change the extent of infarct size (Figure 3e). Immunohistochemical analysis of cross sections revealed that at 4 weeks after MI, the transplanted PKH26‐labelled cycling‐competent CPCs had increased expression of sarcomeric proteins, α‐actinin, as well as the endothelial lineage marker vWF, evidencing their differentiation into cardiomyocyte‐like precursor cells and endothelial cells, respectively (Figure 3f; Supporting Information Figure S8a,b). Limited or no differentiation was evident in the infarcted/border zone of the hearts injected with PKH26‐labelled senescent‐CPCs and non‐CPC c‐kit^neg^ cells (Figure 3f). To determine whether the transplanted cells had participated in inducing a paracrine effect, the infarct/border zone of hearts that had received cells were analysed for formation of new cells that were BrdU‐positive/PKH26‐negative. Hearts that were injected with cycling‐competent CPCs showed an increased number of BrdU‐positive cells, compared to those injected with SA‐β‐gal‐positive senescent‐CPCs or non‐CPC c‐kit^neg^ cells (Supporting Information Figure S8c,d). Moreover, these BrdU‐positive cells co‐localized with vWF or α‐sarcomeric actinin, indicating that these endothelial (capillary) cells and cardiomyocytes had been prompted to initiate DNA synthesis and suggest they may have entered the cell cycle. BrdU‐positive cardiomyocyte and capillaries were more evident (*p* < 0.05) in the MI‐cycling‐competent CPC group (Figure 3g). These findings show the diminished regenerative and reparative capacity of senescent CPCs, compared to healthy, cycling‐competent CPCs.

### 2.4 Aged‐senescent CPCs have a senescence‐associated secretory phenotype (SASP)

**Figure 4:**
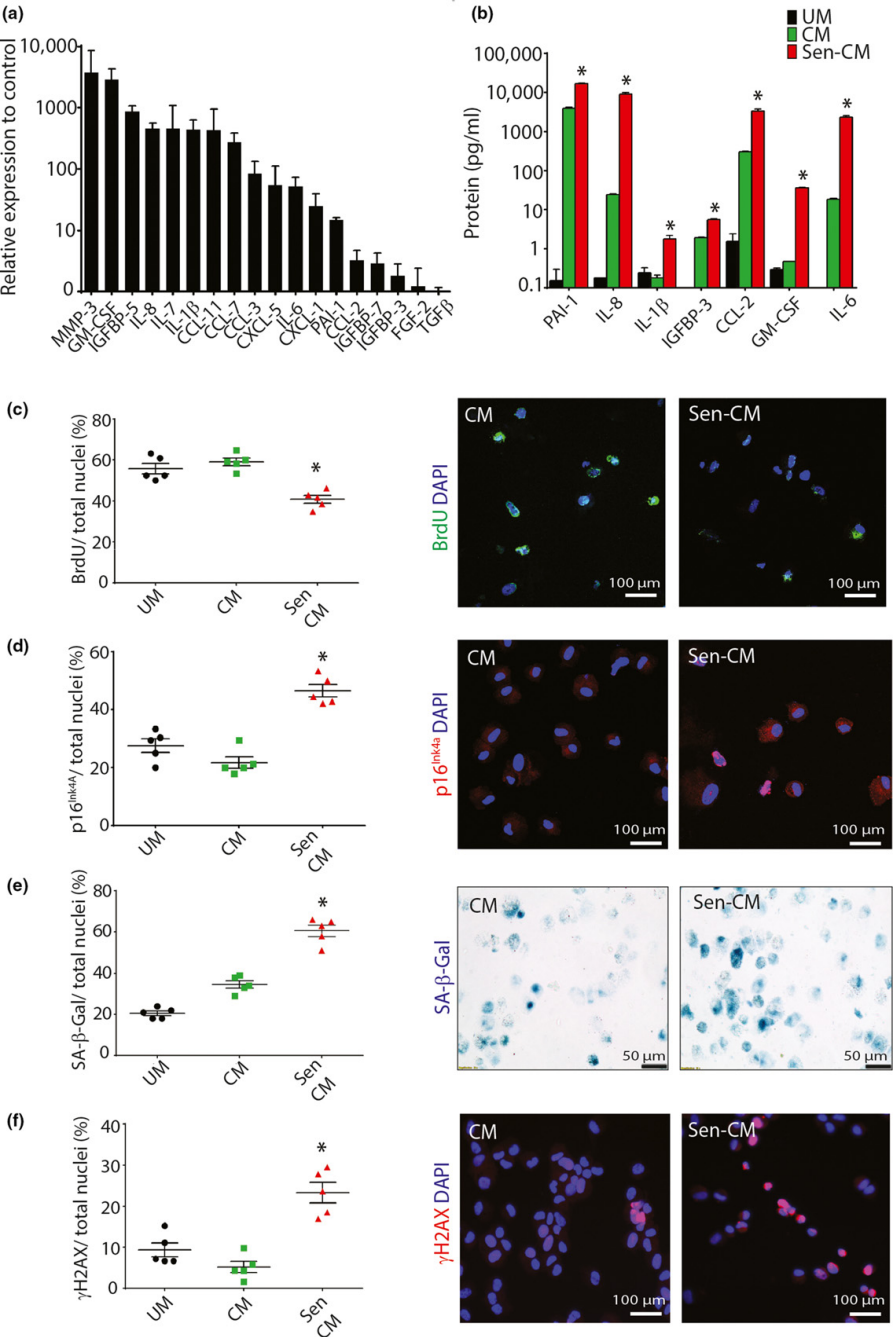
Aged‐senescent CPCs have a SASP (a) Transcript SASP factor expression of dox‐induced Sen‐CPCs relative to Cycling‐CPCs (control). (b) SASP factor protein levels quantified by Luminex of unconditioned media (UM), Cycling‐CPC (CM) and dox‐induced Sen‐CPC (Sen CM) conditioned media (*p < 0.05 vs. UM and CM). Conditioned media applied to cycling‐competent CPCs and the following analyses performed; (c) Quantification and representative staining of CPC proliferation (*p < 0.001; *n* = 5 replicates), (d) p16^INK4A‐pos ^CPCs (*p < 0.0001; *n* = 5 replicates), (e) SA‐β‐gal^pos ^CPCs (*p < 0.0001; *n* = 5 replicates), (f) γH2AX^pos^ CPCs (*p < 0.001; *n* = 5 replicates). All data are mean ± *SEM*

Senescent cells exhibit a SASP (Tchkonia et al., 2013). Dox‐induced senescent SA‐β‐gal‐positive CPCs showed increased expression of SASP factors, including MMP‐3, PAI1, IL‐6, IL‐8, IL‐1β and GM‐CSF, compared to nonsenescent, SA‐β‐gal‐negative, cycling‐competent CPCs (Figure 4a). To determine whether the SASP factors were secreted from senescent‐CPCs, we quantified the protein levels of seven of the highly expressed SASP factors in conditioned media from senescent‐CPCs using Luminex technology. We found increased (*p* < 0.05) quantities of all seven SASP factors in senescent‐CPC conditioned medium, compared to conditioned medium of cycling‐competent CPCs (Figure 4b). Next, we treated cycling‐competent CPCs with conditioned medium (CM) from senescent‐CPCs and measured cell proliferation and senescence of the cycling‐competent CPCs. Conditioned medium from senescent‐CPCs resulted in decreased (*p* < 0.05) proliferation (Figure 4c) and an increased (*p* < 0.05) proportion of senescent p16^INK4A^–positive, SA‐β‐gal‐positive and γH2AX‐positive CPCs in the cultures, compared to CPCs treated with CM from cycling‐competent CPCs or unconditioned medium (UM) (Figure 4d–f). These findings show that the senescent‐CPCs exhibit a SASP, which can negatively impact surrounding cells, rendering otherwise healthy, cycling‐competent CPCs to lose proliferative capacity and switch to a senescent phenotype. This is consistent with the spread of senescence to recipients’ cells that we observed after transplanting another senescent progenitor cell type, senescent adipocyte progenitors (Xu et al., 2018).

### 2.5 Elimination of senescent CPCs using senolytic drugs abrogates the SASP in vitro

**Figure 5:**
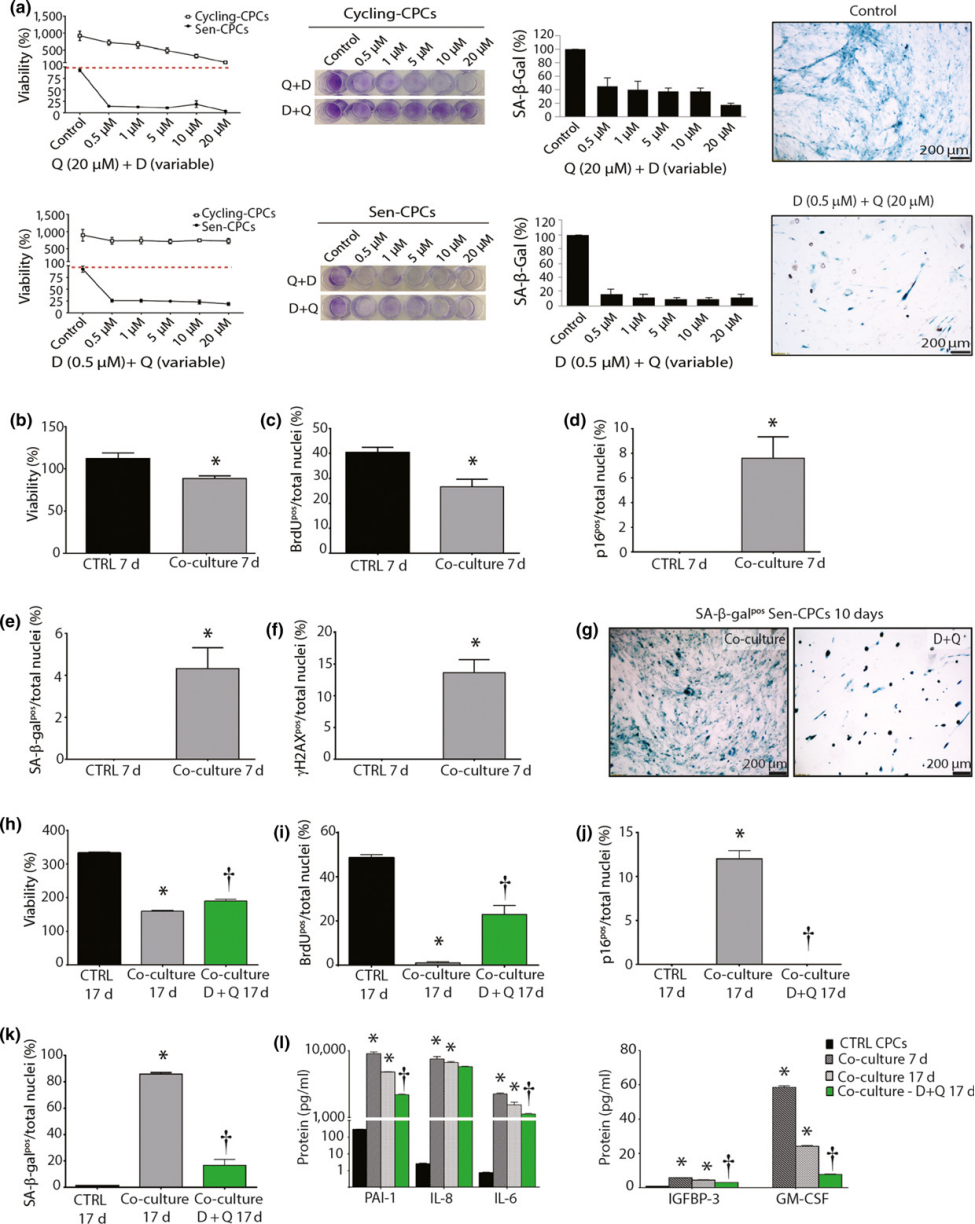
Senolytic clearance abrogates the SASP (a) Viability (Crystal violet) and SA‐β‐gal quantification of dox‐induced Sen‐CPCs and Cycling‐CPCs exposed to various concentrations of D + Q for 3 days. (b–f) Quantification of 7 days of co‐culture of Cycling‐CPCs with dox‐induced Sen‐CPCs for (b) viability (*p = 0.0068); (c) proliferation (*p = 0.0056); (d) p16^INK4A ^(*p = 0.0025); (e) SA‐β‐gal (*p = 0.0025); (f) γH2AX (*p = 0.0002). CTRL is Cycling‐ CPCs alone. (*n* = 5 replicates). (g) Representative SA‐β‐gal staining after clearance of dox‐induced Sen‐CPCs from co‐culture by D + Q treatment. (h–k) Quantification of 17 days of co‐culture of Cycling‐CPCs with dox‐induced Sen‐CPCs or co‐culture with D + Q treatment for (h) viability (*p < 0.0001 vs. CTRL 17 days; †*p* = 0.0001 vs. co‐culture 17 days); (i) proliferation (*p < 0.0001 vs. CTRL 17 days; †*p* < 0.0001 vs. co‐culture 17 days); (j) p16^INK4A ^(*p < 0.0001 vs. CTRL 17 days; †*p* < 0.0001 vs. co‐culture 17 days) and (k) SA‐β‐gal (*p < 0.0001 vs. CTRL 17 days; †*p* < 0.0001 vs. co‐culture 17 days). CTRL is Cycling‐CPCs alone. (*n* = 5 replicates). (l) SASP factor protein levels quantified by Luminex from each treatment condition (*p < 0.0001 vs. CTRL; †*p* < 0.01 vs. co‐culture 7 days and 17 days). (*n* = 2 replicates). All data are mean ± *SEM*

Removal of p16^Ink4a^ senescent cells can delay the acquisition of age‐related pathologies in adipose tissue, skeletal muscle, heart, blood vessels, lung, liver, bone and eye (Baker et al., 2016, 2011; Farr et al., 2017; Lehmann et al., 2017; Ogrodnik et al., 2017; Roos et al., 2016; Schafer et al., 2017; Xu, Palmer, et al., 2015; Xu, Tchkonia, et al., 2015; Xu et al., 2018; Zhu et al., 2015). Recent studies have documented the use of senolytic drugs for the selective clearance of senescent cells from “aged” tissues (Tchkonia & Kirkland, 2018). We tested the potential of four senolytic drugs, Dasatinib (D; an FDA‐approved tyrosine kinase inhibitor), Quercetin (Q; a flavonoid present in many fruits and vegetables), Fisetin (F; also a flavonoid) and Navitoclax (N; an inhibitor of several BCL‐2 family proteins), alone and in combination to eliminate and clear senescent‐CPCs in vitro (Supporting Information Figure S9). Measuring cell viability with crystal violet and the number of SA‐β‐gal‐positive CPCs, dose–response experiments on senescent‐ or cycling‐competent CPCs from the same subjects showed D and N to effectively clear senescent‐CPCs, whereas F and Q were less effective (Supporting Information Figure S9b,c). However, D also decreased the viability of cycling‐competent CPCs (Supporting Information Figure S9b). A combination of D + Q, which has previously shown to yield effective senescent cell clearance (Farr et al., 2017; Lehmann et al., 2017; Ogrodnik et al., 2017; Roos et al., 2016; Schafer et al., 2017; Xu et al., 2018; Zhu et al., 2015) and which does not share the toxic anti‐neutrophil and anti‐platelet side effects of N (Kirkland & Tchkonia, 2015; Kirkland & Tchkonia, 2017) was tested, and at a dose of 0.5 µM D with 20 µM Q, cycling‐competent CPC viability was preserved (Figure 5a) while senescent‐CPCs were cleared and induced to selective apoptosis (Supporting Information Figure S9d).

We next determined whether clearing senescent‐CPCs using D + Q would abrogate the SASP and its paracrine impact on CPCs. Using transwell inserts, cycling‐competent CPCs were seeded on the top chamber insert and co‐cultured in the presence of senescent‐CPCs seeded on the bottom chamber. Cultures were left for 7 days, and then cycling‐competent CPCs in the top chamber were analysed for viability, proliferation and markers of senescence, p16^INK4A^, SA‐β‐gal and γH2AX, and conditioned medium analysed for SASP factors. The cultures were then treated with D + Q for 3 days to clear the senescent‐CPCs on the bottom chamber, and then 7 days later cycling‐competent CPCs in the top chamber were analysed for viability, proliferation and the markers of senescence, p16^INK4A^, SA‐β‐gal and γH2AX, and conditioned medium analysed for SASP factors (total of 17 days; Supporting Information Figure S9a). We found that cycling‐competent CPCs co‐cultured in the presence of senescent‐CPCs for 7 days were decreased (*p* < 0.05) in number and proliferation and had increased (*p* < 0.05) expression of p16^INK4A^, SA‐β‐gal and γH2AX (Figure 5b–f). Application of D + Q to co‐cultures eliminated the senescent‐CPCs (Figure 5g) and 7 days later, the cycling‐competent CPCs had increased (*p* < 0.05) in number (Figure 5h), proliferation (Figure 5i), and the number of p16^INK4A^ and SA‐β‐gal CPCs had decreased (*p* < 0.05) compared to CPCs that had been in co‐culture with senescent‐CPCs for 17 days (Figure 5j,k). Co‐culture of cycling‐competent CPCs with senescent‐CPCs led to increased (*p* < 0.05) secretion of SASP factors into the medium, but the level of SASP factors was reduced (*p* < 0.05) with application of D + Q (Figure 5l). These findings document that senescent CPCs have a SASP, and clearance of senescent CPCs using a combination of D + Q senolytics abrogates the SASP and its detrimental senescence‐inducing effect on healthy, cycling‐competent CPCs.

### 2.6 Elimination of senescent cells in vivo activates resident CPCs and increases number of small, proliferating cardiomyocytes in the aged heart

**Figure 6:**
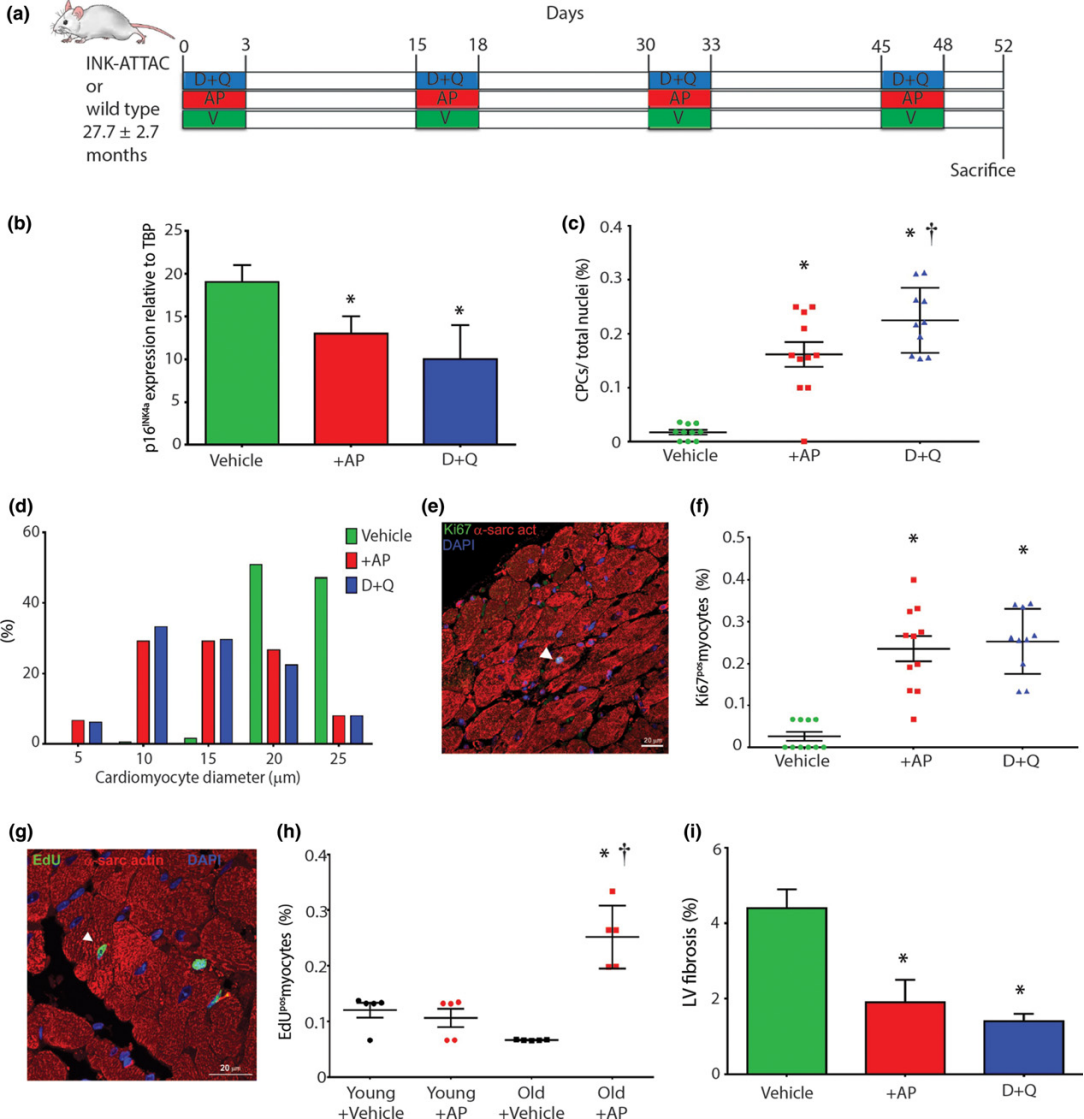
Clearance of senescent cells stimulates new cardiomyocyte formation in the aged heart (a) In vivo senescent cell clearance experimental design. (b) Total cardiac p16^Ink4a^ gene expression following clearance (*p < 0.01 vs. Vehicle; *n* = 5 mice). (c) Quantification of CPCs following clearance, (*p < 0.0001 vs. Vehicle; †*p* = 0.0453 vs. AP; *n* = 10–11 mice). (d) Frequency distribution histogram of cardiomyocyte diameter, (*n* = 6 mice). (e) A Ki67^pos^/α‐sarcomeric actin^pos^ cardiomyocyte (arrowhead) in the LV of a 32 month D + Q‐treated mouse. (f) Quantification of Ki67^pos ^cardiomyocytes, (*p < 0.0001 vs. Vehicle; *n* = 10 mice). (g) An EdU^pos^/α‐sarcomeric actin^pos^ cardiomyocyte in the LV of a 22 month INK‐ATTAC AP‐treated mouse. Nuclei are stained by DAPI in blue. (h) Quantification of EdU^pos ^cardiomyocytes (*p < 0.0001 vs. Old + Vehicle; †*p* < 0.0001 vs. Young + AP; *n* = 5 mice). (i) Quantification of LV fibrosis (*p < 0.05 vs. Vehicle; *n* = 3 mice). All data are mean ± *SD*

To determine the effects of global senescent cell removal on the heart, 24–32 month INK‐ATTAC transgenic or wild‐type mice were randomised to either vehicle, AP20187, or D + Q treatment, administered in 4 cycles for 3 consecutive days/cycle, 12 days apart. Mice were sacrificed 4 days after the last dose of cycle 4 (Figure 6a). Tissues in which p16^Ink4a^ expression and/or senescent cells are decreased by D + Q in wild‐type mice as well as by AP20187 in INK‐ATTAC mice include the aorta, adipose tissue, cardiac and skeletal muscle, lung, liver and bone (Baker et al., 2016; Farr et al., 2017; Lehmann et al., 2017; Ogrodnik et al., 2017; Roos et al., 2016; Schafer et al., 2017; Xu et al., 2018; Zhu et al., 2015). We showed p16^Ink4a^ mRNA expression was decreased (*p* < 0.05) in the heart following D + Q or AP20187 treatment in aged INK‐ATTAC or wild‐type mice (Figure 6b).

Previously, we have shown an improvement of heart function in old mice after D + Q treatment (Zhu et al., 2015). Analysis of cardiac cross sections revealed significantly higher (*p* < 0.05) CPC (Sca‐1^+^/c‐kit^+^/CD45^−^/CD31^−^/CD34^−^) numbers (Figure 6c; Supporting Information Figure S10a) in AP20187‐treated INK‐ATTAC mice and D + Q‐treated INK‐ATTAC or wild‐type mice, compared to vehicle‐treated control. Interestingly, D + Q treatment showed increased (*p* < 0.05) CPC number, compared to AP20187‐treatment (Figure 6c). Roughly 10% of CPCs were activated and in the cell cycle (Ki67‐positive) at the time of sacrifice, after AP20187 or D + Q treatment (Supporting Information Figure S10b). Morphometric analysis of heart sections showed that AP20187‐treated and D + Q‐treated mice had increased number of smaller ventricular myocytes (Figure 6d), suggesting these myocytes to be immature and newly formed, compared to vehicle‐treated mice, which exhibited only rare small myocytes but a greater proportion of hypertrophied myocytes (Figure 6d). We found an increase (*p* < 0.05) of small, proliferating Ki67‐positive myocytes (~0.25%) in old hearts following AP20187‐ or D + Q‐treatment, compared to vehicle‐treated control (0.03 ± 0.03%) (Figure 6e,f). To corroborate these data, we injected EdU 4 days and 2 hr prior to sacrifice of old (22 months) and young (3 months) AP20187‐treated INK‐ATTAC mice (Supporting Information Figure S10c). We found increased (*p* < 0.05) number of small EdU‐positive myocytes (0.25 ± 0.06%; Figure 6g,h) in the hearts of old AP‐treated mice, compared to old vehicle‐treated (0.07 ± 0.00%), young vehicle‐treated (0.12 ± 0.03%) and young AP‐treated (0.11 ± 0.04%) mice. The number of EdU‐positive myocytes in the old AP‐treated INK‐ATTAC mice was the same in amount to Ki67‐positive myocytes (~0.25%) in AP20187‐ or D + Q‐treated mouse hearts (Figure 6f). Finally, we detected a decrease (*p* < 0.05) in fibrosis in the LV following AP20187‐ and D + Q‐treatment, compared to vehicle‐treated control (Figure 6i). In contrast to the treatment of aged mice, treatment of young adult (2–3 months) INK‐ATTAC or wild‐type mice with AP20187 or D + Q, respectively, did not alter EdU‐positive myocyte number (Figure 6h), CPC numbers or myocyte diameter (*data not shown*). These findings show that clearance of senescent cells leads to stimulation of CPCs and cardiomyocytes with increased DNA‐synthesizing activity and that this strategy is specific to the aged heart.

## 3 DISCUSSION

Our study shows that CPCs isolated from the failing human heart develop a senescent phenotype with age exhibited by increased expression of senescence‐associated markers (p16^INK4A^, SA‐β‐gal), DNA damage, shortened telomere length and a SASP. Aged human hearts with dilated cardiomyopathy showed greater numbers of p16^INK4A ^–positive CPCs and cardiomyocytes with shorter telomeres than age‐matched controls (Chimenti et al., 2003). Similarly, CPCs isolated from failing, aged hearts show increased p16^INK4A ^and inflammatory factor expression (Cesselli et al., 2011). Reliably detecting senescent cells in vivo is an ongoing challenge, and it is important that a combination of senescent cell biomarkers is used for detection as any one marker used in isolation is prone to false positives. Our study used a combined panel of the senescence‐associated biomarkers, p16^INK4A^, γH2AX, telomere length, SA β‐gal activity and SASP expression, to detect senescent CPCs. A limitation of our study is that because of low yield of CPCs isolated from small myocardial samples (~45,000 per gram of tissue) we were unable to evaluate the expression of SASP factors on freshly isolated CPCs, or test the effects of the SASP of freshly isolated CPCs in vitro. Our findings demonstrate that CPCs accumulate in the failing hearts of elderly subjects (>70 years) and are dysfunctional, showing impaired proliferation, clonogenicity, spherogenesis and differentiation, compared to CPCs isolated from the hearts of middle‐aged (32–66 years) subjects. As the adult heart possesses very low numbers (<1% of c‐kit^pos^ cells) of cardiomyogenic CPCs (Vicinanza et al., 2017), if by 80 years of age >50% of resident CPCs are senescent, this presents a bleak outcome for harvesting healthy, functional CPCs from patients who are candidates for regenerative therapies and their autologous use. Moreover, strategies to activate the regenerative capacity of the aged heart through delivery of growth factors or cell therapy will also be suboptimal. Therefore, the success of cardiac regenerative therapeutic approaches thus far tested for treating patients with heart failure and disease could be of limited efficacy in promoting myocardial regeneration because of the increased number of senescent, dysfunctional CPCs and cardiomyocytes (Cesselli et al., 2011; Chimenti et al., 2003), and the resultant presence of a cardiac SASP in the aged and failing heart that impairs the function of the remaining nonsenescent CPCs. These findings may also have implications regarding the use hearts from aged versus younger donors for transplantation (Lau, Kennedy, Kirkland, Tullius, & S.G., 2019).

The present study found that CPCs age in a stochastic nonautonomous manner, and it is possible to clonally select from a single CPC for a cycling‐competent population of CPCs even from diseased or aged hearts. There are individual CPCs in older individuals that have replicative and functional capacities resembling those of CPCs in younger subjects. A similar scenario was found in the case of rat fat cell progenitors (Kirkland et al., 1990). While the abundance of progenitors cloned from adipose tissue that had restricted capacities for replication and differentiation into adipocytes or that were nonreplicative but viable (i.e., senescent) increased progressively with aging in rat adipose, there remained cells that had the capacities for replication and adipogenic differentiation characteristic of clones derived from young rats. In the present study, we utilized hypoxic conditions (5% CO_2_, 2% O_2_) to expand viable CPCs. Other interventional approaches have shown that senescence characteristics of human CPCs are alleviated by Pim‐1 kinase resulting in rejuvenation of CPC phenotypic and functional properties (Mohsin et al., 2013). Together, these findings indicate that: (a) it may be feasible to isolate and propagate CPCs even from older individuals that are functional, capable of supporting cardiac regeneration if removed from their toxic *milieu*, and that could be therapeutically relevant in treating patients, especially if they were autologously generated and (b) that by clearing senescent CPCs with a toxic SASP from the aged heart, there remains a tissue‐resident population of CPCs with regenerative and reparative potential.

When we purified for a homogenous SA‐β‐gal‐positive, senescent CPC population, we showed that these cells had poor engraftment and survival and were unable to contribute to cardiac regeneration, repair or restoration of cardiac function following transplantation into the infarcted myocardium. This is contrary to in vitro‐selected, SA‐β‐gal‐negative, proliferative cycling‐competent (Ki67^pos^) CPCs, which had high survival and engraftment in the infarct/border zone, restored cardiac function almost to baseline and sham control values (LVEF 59 ± 2% at 28 days vs. 66 ± 2 at baseline), decreased infarct size, differentiated into endothelial and cardiomyocyte‐like precursor cells as well as enhanced endogenous BrdU^pos ^cardiomyocytes and capillaries. Although some of the transplanted CPCs expressed α‐sarcomeric actin, these cells did not exhibit the typical cardiomyocyte phenotype as they were small and lacked a structured sarcomeric unit. Therefore, they could not be considered as new, immature myocytes that contributed physiologically to the substantially improved LV function. There is now a general consensus that the favourable effect of cell transplantation protocols is, at least in part, mediated by “paracrine” effectors secreted by the transplanted cells, contributing to improved myocardial contractility and amelioration of ventricular remodelling (decreasing fibrosis, hibernation, and stunning), inhibition of the inflammatory response, and increased cardiomyocyte survival, cardiomyogenesis and angiogenesis/neovascularisation (Broughton et al., 2018). The present data emphasize the importance of taking into account the hostile infarcted environment, which does not favour engraftment, differentiation or maturation of newly formed cardiomyocytes derived from injected cells. However, the presence of CPC‐derived cardiomyocyte precursors expressing sarcomeric protein in the infarct/border zone is promising, and further work should elucidate how to mature these cells into functionally competent contractile cells.

Like the present study, not all experimental studies have shown physiological regeneration of CPC‐derived cardiac muscle following administration of CPCs (Tang et al., 2016). This is most likely due to the heterogeneous nature of cardiac c‐kit positive cells tested, with only a very small fraction (1%–2%) having properties of cardiomyogenic stem cells (Vicinanza et al., 2017). However, bringing together the cardiomyogenic potential of CPCs, which can be amplified in number through in vitro single cell‐derived clonal selection, and CPC‐mediated cytokine release, advocate CPCs as an ideal candidate for cardiac regenerative interventions.

Senescent cells have emerged as bona fide drivers of aging and age‐related CVD, which suggests strategies aimed at reducing or eliminating senescent cells could be a viable target to treat and prevent CVD (Childs, Li, & Deursen, 2018). In 18‐month‐old INK‐ATTAC mice, p16^INK4a^‐positive cells contribute to cardiac aging and these senescent cells decreased following AP20187‐treatment (Baker et al., 2016). Moreover, D + Q administration over 3 months decreased senescent cell markers (TAF + cells) in the media layer of the aorta from aged (24 months) and hypercholesterolemic mice, which was met with improved vasomotor function (Roos et al., 2016). We show for the first time that the genetic and pharmacological approaches used here to reduce senescent cell burden leads to increased number of CPCs and smaller ventricular myocytes, which were Ki67‐positive and EdU‐positive, suggesting these myocytes to be immature and newly formed (Ellison et al., 2013), compared to vehicle‐treated mice, which exhibited very rare small myocytes but a greater proportion of hypertrophied myocytes. These findings are in line with those of Baker et al. (2016) who showed that AP‐treated INK‐ATTAC mice had smaller ventricular cardiomyocytes.

The frequency of resident cardiac stem and progenitor cells in the healthy myocardium of several mammalian species, including human, mouse, rat and pig, is approximately one per every 1,000–2,000 myocytes, depending on age (Torella, Ellison, Karakikes, & Nadal‐Ginard, 2007). We detected a 16‐ and 23‐fold increase in the number of CPCs following elimination of senescent cells by AP‐ or D + Q‐treatment in the aged mouse heart, respectively. Moreover, ~10% of CPCs after elimination of senescent cells were activated, expressing Ki67. The number of Ki67‐positive and EdU‐positive cardiomyocytes increased nine‐ and four‐fold in the aged heart, respectively, following clearance of senescent cells by either D + Q‐ or AP‐treatment. We show that the number of DNA‐synthesizing cardiomyocytes present in the young (3 months old) mouse heart is 0.12 ± 0.03% of total cardiomyocytes, and the number of DNA‐synthesizing cardiomyocytes present in the old (22 months old) mouse heart is 0.07 ± 0.00% of total cardiomyocytes. Elimination of senescent cells lead to double the amount of DNA‐synthesizing cardiomyocytes found in a young heart and triple the number found in an old heart. Therefore, the present data represent a significant and physiologically relevant increase and activation of the resident CPC compartment and increased cardiomyocyte DNA synthetic activity following clearance of senescent cells. These findings are consistent with the hypothesis that clearing senescent cells can beneficially alter stem and progenitor cell function across multiple tissues. D + Q enhances function of osteoblastic progenitors, leading to new bone formation in mice with age‐related osteoporosis, impedes function of the osteoclast progenitors that lead to bone resorption in these same mice (Farr et al., 2017), and enhances neurogenesis in mice with metabolically induced impairment of nerve cell generation (Ogrodnik et al., 2019).

Previous work has shown that senescent human primary preadipocytes, human omental adipose tissue cells obtained from obese individuals (45.7 ± 8.3 years), as well as human umbilical vein endothelial cells (HUVECs) develop a SASP with aging, which induced inflammation in healthy adipose tissue and preadipocytes (Xu, Palmer, et al., 2015; Xu, Tchkonia, et al., 2015; Xu et al., 2018). Clearance of senescent cells using D + Q decreased the secretion of key SASP components, PAI‐1, GM‐CSF, IL‐6, IL‐8 and MCP‐1 (Xu et al., 2018). In the present study, clearance of senescent human CPCs using D + Q abrogated the SASP and the deleterious impact of the SASP on CPC proliferation and inducing senescence. Thus, D + Q can kill human senescent cells, including tissue‐specific stem/progenitor cells, and can attenuate the secretion of inflammatory cytokines associated with human age‐related frailty (Xu et al., 2018). Consistent with this, in the first human trial of senolytics, subjects with idiopathic pulmonary fibrosis had enhanced walking endurance, gait speed, chair rise test performance and scores in the Short Physical Performance Battery 5 days after nine doses of D + Q over 3 weeks (Justice et al., 2019).

In conclusion, the present work, albeit performed in mouse models, demonstrates approaches that eliminate senescent cells may be useful for treating age‐related cardiac deterioration and rejuvenating the regenerative capacity of the aged heart. However, caution should be exercised in interpreting mouse data considering previous cardiac regenerative strategies where bone marrow derived cells injected into mouse MI models did not translate into equivalent outcomes in humans. Future work should address the effects of senolytic agents on improving cardiac function and alleviating the SASP, resulting in an improved microenvironment in vivo and the activity of other cell types, such as fibroblasts and endothelial cells. Indeed, next steps should determine whether senolytic approaches could be used in conjunction with cell therapy interventions to improve the environment (the “soil”) that the cells (the “seeds”) are transplanted into and therefore, ameliorate intrinsic reparative mechanistic processes that are compromised with age (Lau et al., 2019). Indeed, targeting senescent cells could also impact the potency of resident stem/progenitor populations in other aged organs. The present findings provide new insights into therapies that target senescent cells to prevent an age‐related loss of regenerative capacity.

## 4 EXPERIMENTAL PROCEDURES

### 4.1 Expanded experimental procedures are in the supporting information

Myocardial samples (~200 mg each) were obtained from the right atrial appendage (total samples, *n* = 119) of human subjects with cardiovascular disease, aged 32–86 years. Subjects aged 70–86 years were included in the old group. Subjects aged 32–67 years were included in the middle‐aged group. All subjects gave informed consent before taking part in the study (NREC #08/H1306/91). Cardiac tissue was minced and enzymatically digested to release cardiac small cells. CPCs were purified by first depleting CD45^pos^ and CD31^pos^ cells by immunolabelling with anti‐human CD45 and CD31 magnetic immunobeads (Miltenyi), and then the CD45‐ CD31‐depleted fraction was enriched for c‐kit^pos^ cells through incubation with anti‐human CD117 immunobeads (Miltenyi). CPCs were characterized by flow cytometry for other CPC markers before proceeding with functional in vitro and in vivo assays. All animal surgical experiments were conducted in accordance with the regulations of the Home Office and stipulated under the Animals (Scientific Procedures) Act 1986.

## CONFLICT OF INTEREST

None declared.

## AUTHOR CONTRIBUTIONS

F.C.L‐M., P.J.R., E.D‐V., T.S.T., B.J.C. and L.P performed experiments and analysis. J.E.C. and D.T were involved in conceptualization. F.C.L‐M., P.J.R., T.T., G.M.E‐H. and J.L.K were involved in conceptualization, writing and editing. G.M.E‐H., T.T. and J.L.K supervised and performed funding acquisition.
