# Supplementary material for: Aged‐senescent cells contribute to impaired heart regeneration
Source: Aging Cell. 2019 Mar 10;18(3):e12931. doi: 10.1111/acel.12931 (PMC6516154; doi:10.1111/acel.12931)
Supplement: Supplementary file 1 [file ACEL-18-e12931-s001.pdf]

Supporting information file

Methods

Human CPC isolation

Myocardial samples (~200mg each) were obtained from the right atrial appendage of subjects with cardiovascular disease, aged 22-86 years (total subjects included in study, n=119). All subjects had given informed consented to take part in the study (NREC #08/ H1306/91). Samples were stored in saline on ice until ready to process (~1hr). All steps were performed at 4°C unless stated otherwise. Briefly, cardiac tissue was minced then digested with collagenase II (0.3mg/ml; Worthington Laboratories) in Dulbecco's Modified Eagle's Medium (DMEM; Sigma-Aldrich) at 37°C in a series of sequential digestions for 3 minutes each. Enzymatically released cells were filtered through a 40μm cell strainer (Becton Dickinson, BD) and collected in enzyme quenching media (DMEM + 10% FBS). The isolated cardiac cells were collected by centrifugation at 400g for 10 min, resuspended in incubation media (PBS, 0.5% BSA, 2 mM EDTA), and passed through an OptiPrep™ (Sigma-Aldrich) density gradient medium to remove large debris. This involved layering the cardiac cell population on top of 16% and 36% OptiPrep™:DMEM solutions and then centrifuging for 20 min at 800g with no brake. Larger debris collected at the bottom of the tube, allowing for selective retrieval of the small cell fraction from the upper layers. For the isolation of c-kit^{pos}, CD45^{neg}, CD31^{neg} CPCs, first the small cardiac cells were depleted of CD45^{pos} and CD31^{pos} cells by immunolabelling with anti-human CD45 and CD31 magnetic immunobeads (Miltenyi) diluted (1:10) in incubation media for 30 minutes at 4°C with agitation. After antibody binding, the CD45^{pos}/CD31^{pos} cells were depleted from the preparation using magnetic activated cell sorting (MACS; Miltenyi). The elution cells, which were CD45^{neg}, CD31^{neg} were then enriched for c-kit^{pos} cardiac cells through incubation with anti-human CD117 immunobeads (Miltenyi) (1:10) for 30 minutes at 4°C with agitation and again sorted using MACS according to the manufacturer's instructions. Antibodies used for selection are detailed in **Supplementary Table 1**.

Flow cytometry and FACS

The c-kit^{pos}, CD45^{neg}, CD31^{neg} human CPCs were analysed for hematopoietic, mesenchymal, and endothelial cell markers using a FACSCanto II™ flow cytometer (BD) as previously described (Lewis, Henning et al. 2014). The antibodies used for flow cytometry and FACS are reported in **Supplementary Table 1**. Respective isotype controls (Biolegend) were used

as negative controls for all flow cytometry procedures. All antibodies were applied at 1:10 diluted in incubation media for 30 min at 4°C with agitation. Data were analysed using FlowJo® software (FlowJo LLC).

SA–β-gal^{pos} CPC sorting was performed by fluorescent activated cell sorting (FACS) using an ImaGene Green C_{12}FDG lacZ gene expression kit (Life Technologies, USA) according to the manufacturer's instructions and protocols previously reported (Debacq-Chainiaux, Erusalimsky et al. 2009). Briefly, freshly isolated CPCs were resuspended in 1ml of 33µM C_{12}FDG β-gal diluted in pre-warmed DMEM/F12 media and incubated at 37°C for 30 minutes with agitation. The cell suspension was then sorted based upon SA–β-gal^{pos} fluorescence using FACS.

Cell culture

Human CPCs were cultured on CELLstart™ (ThermoFisher Scientific)-coated cultureware in growth medium, which is composed of (1:1) Dulbecco's MEM/Ham's F12 (DMEM/F12; Sigma-Aldrich) and Neurobasal A medium (ThermoFisher Scientific) containing 10% Stemulate® pooled human platelet lysate (Cook medical, USA), 2mM L-alanyl-L-glutamine (ThermoFisher Scientific), B27 and N2 supplements (ThermoFisher Scientific), leukemia inhibitory factor (LIF) (10ng/ml; Millipore), bFGF (10ng/ml; Peprotech), EGF (20ng/ml; Peprotech), insulin-transferrin-selenite (ITS; ThermoFisher Scientific), 1% penicillin/streptomycin (ThermoFisher Scientific), 0.1% gentamicin (ThermoFisher Scientific), and 0.1% Fungizone™ (ThermoFisher Scientific) in a humidified hypoxic incubator at 37°C, 5% CO_2, 2% O_2.

Immunocytochemistry

Human CPCs were either freshly isolated or grown in culture and then directly cytospun onto poly-lysine-coated slides using a Shandon Cytospin 4 Cytocentrifuge (ThermoFisher Scientific). Slides were immediately fixed using Shandon Cell-Fixx (ThermoFisher Scientific). After fixation, cells were allowed to air dry before proceeding with immunostaining. To prepare cells for immunostaining, slides were incubated with 95% ethanol for 15 min at room temperature, washed with PBS, and in the case of intracellular staining, incubated in 0.1% Triton X-100:PBS at room temperature for 10 minutes. After washing with 0.1% tween:PBS and a 1 hr incubation in 10% donkey serum, cells were incubated overnight at 4°C with primary antibodies to c-kit, CD45, CD31, CD34, p16^{INK4A},

γH2AX, or Ki67 (**Supplementary Table 2**) all applied at 1:50 in 0.1% Tween:PBS. Slides were then washed and incubated with corresponding Alexafluor™ secondary antibodies (Life Technologies) for 1 hour at 37°C. The nuclear DNA of the cells was counterstained with 4',6-diamidino-2-phenylindole (DAPI) (Sigma-Aldrich) at 1µg/ml and mounted using Vectasheild mounting media (Vector labs). The cells were viewed and imaged using an ApoTome fluorescent microscope (Zeiss) or A1 confocal microscope (Nikon). A minimum of 20 random fields of view at x20 magnification were used to determine the percentage of positively stained cells, expressed as a percent of total nuclei.

SA–β-gal (SABG) staining was performed using a SA–β-gal staining kit (Cell Signaling Technology) according to the manufacturer's instructions and protocols previously reported (Debacq-Chainiaux, Erusalimsky et al. 2009).. In brief, human CPCs were either freshly isolated and directly cytospun onto poly-lysine-coated slides (ThermoFisher Scientific) or grown in culture and then fixed *in situ* using 2% paraformaldehyde: PBS (vol/vol) (Sigma-Aldrich) for 5 minutes at room temperature. Following fixation, cells were washed with PBS before being incubated in SABG activity solution (pH 6.0) at 37°C overnight. The enzymatic reaction was stopped by washing slides with ice-cold PBS and SABG staining was fixed with ice-cold methanol for 30s before mounting/visualising. Senescent CPCs were identified as blue-stained cells using light microscopy. A minimum of 10 images were taken at x4 magnification from random fields and the percentage of SA–β-gal cells were expressed as percentage of total nuclei.

Q-FISH analysis

Freshly isolated human CPCs were cytospun and fixed as described above. To perform Q-FISH, slides were prepared using a telomere PNA FISH kit (Dako) according to the manufacturer's instructions. In brief, slides were incubated in 95% ethanol for 5 min and washed in Tris-buffered saline (TBS; 2 min), fixed in formaldehyde (3.7%) in PBS (2 min), washed in TBS (2 x 5 min), and a pre-treatment solution applied at 1:2000 in TBS (10 min). Slides were washed again with TBS (2 x 5 min), dehydrated in cold ethanol series (70%, 80%, 95%, 2 min each), and air dried. The PNA hybridization mixture (10µl) containing Cy-3-conjugated $(C_3TA_2)_3$ peptide nucleic acid (PNA) probe was added to the cells, a coverslip (18 × 18 mm) was then applied, followed by DNA denaturation (5 min at 80°C). After hybridization for 30 min at room temperature, slides were incubated in wash solution with agitation (5 min at 65°C). Slides were counterstained with DAPI (Sigma-Aldrich) and

mounted using Vectashield mounting media (Vector labs). All steps were performed at room temperature unless otherwise stated. Images were captured at 100x magnification on an A1 confocal microscope (Nikon). To prevent a possible selection bias, images were acquired ‘blindly’ *i.e.*, cells were chosen solely on the basis of DAPI staining without checking the corresponding Cy-3 image. Telomere fluorescence signals were measured from 20 single cells per sample using ImageJ software (Fiji, USA). Cells and telomeres were identified through segmentation of the DAPI image and the Cy-3 image, respectively. The integrated fluorescence intensity for each telomere was calculated after correction for background, based on the values of the surrounding pixels. Telomere fluorescence values were converted into Kb by calibration with the L5178-Y and L5178Y-R mouse lymphocyte cell lines with known telomere lengths of 10.2 and 79.7 kb, respectively (McIlrath, Bouffler et al. 2001).

Proliferation, clonogenicity, cardiosphere formation and cardiomyocyte differentiation assays *in vitro*

A BrdU incorporation assay (Roche) was used to determine cell proliferation, as previously described (Ellison, Torella et al. 2011).. In brief, 2.5 x 10^3 CPCs were plated in 24 well plates and serum-starved for 6 h in DMEM/F12 medium. Medium was then replaced with growth medium and BrdU was added, 1μg/ml every 8 hours. Cells were fixed after 24 hours and BrdU incorporation was assessed using a BrdU detection kit (Roche) according to the manufacturer’s instructions (**Supplementary Table 2**). Nuclei were counterstained with DAPI (Sigma-Aldrich). Cells were viewed and imaged using an ApoTome fluorescent microscope (Zeiss). A minimum of 10 random fields of view at x20 magnification were analysed and number of BrdU-positive cells expressed as a percent of total nuclei.

Human CPCs (P2-P3) were serially diluted and deposited into CTS CELLstart pre-coated 96-well plates (Corning Inc., USA) at 1 cell per well, as described previously (Vicinanza, Aquila et al. 2017). Individual cells were cultured in growth medium which was refreshed every 3 days. Wells containing CPC colonies were scored by bright-field microscopy after 7 days of culture to determine clonal efficiency. The clonogenicity of the human CPCs was expressed as the percent number of clones generated from the total number of single cells plated. A minimum of 3 plates were quantified per subject.

Cardiomyocyte differentiation was induced as previously described (Smith, Lewis et al. 2014, Vicinanza, Aquila et al. 2017). Briefly, 5x10^4 human CPCs were treated with oxytocin (100

nM; Sigma-Aldrich) for 72 h. Human CPCs were then transferred to a 10cm bacteriological dish containing LIF–deprived CPC growth medium to generate spheres. Spheres were transferred to laminin (1μg/ml; Sigma-Aldrich)-coated coverslips within 24 well plates and incubated with cardiomyogenic differentiation medium: alpha-MEM, 2% FCS, ascorbic acid (50μg/ml), dexamethasone (1 μM), beta-glycerol phosphate (10 mM), 1% penicillin-streptomycin, 0.1% gentamicin, and 0.1% Fungizone. Differentiating cultures were maintained for up to 14 d, replacing the medium containing specific growth factors on days 1 and 5. From days 1–4, cells were incubated with medium containing BMP-2 (10 ng/ml; Peprotech), BMP-4 (10 ng/ml; Peprotech), and TGF-β1 (5 ng/ml; Peprotech). From days 5 to 14, cells were incubated with medium containing 150 ng/ml Dkk-1 (150ng/ml; R&D). Medium was replaced every 3 days and cultures were fixed and analysed at 14 days. Cardiosphere formation was quantified from a minimum of 10 images per subject taken at x4 magnification from random fields. The number of cardiospheres were expressed per cm^2. Cardiosphere size was determined from the same images by measuring the diameter of the spheres using ImageJ software (Fiji, USA). A minimum of 20 spheres per donor were measured. Cardiomyocyte differentiation was measured by immunostaining of differentiated cultures using Nkx2.5 (1:20, overnight 4°C) and α-sarcomeric actin (1:50, 1 hour 37°C) antibodies (**Supplementary Table 2**). Secondary antibodies were Alexa Fluor 488, or 594 (Life Technologies). Nuclei were counterstained with DAPI. A minimum of 5 images at x20 magnification from random fields were taken per subject for quantification of Nkx2.5^{pos} cells per total nuclei and fluorescent intensity of α-sarcomeric actin expression.

Senescence induction, conditioned media, and multiplex SASP protein analysis

To induce senescence pharmacologically, cycling-competent CPCs were exposed to Rosiglitazone (0.1 μM; Calbiochem) or Doxorubicin (0.2 μM; Calbiochem) at ~70% confluence for 24 h. Medium was removed and cell cultures washed three times with PBS before replacing with fresh growth medium. Culture medium was refreshed every 3 days and CPCs were maintained for up to 35 days. Cells were analysed for SA-β-gal (as described above) and p16^{INK4A} expression over this time course by quantifying the total number of positive cells per total nuclei from a minimum of 5 random fields at 10x and 20x magnification respectively. Conditioned media (CM) were prepared by pre-washing cycling-competent or senescent CPC cultures ($5x10^5$ cells) three times with PBS, then exposing them to RMPI 1640 containing 1 mM sodium pyruvate, 2 mM glutamine, MEM (minimum essential medium), vitamins, MEM non-essential amino acids, and antibiotics (ThermoFisher

Scientific) for 24 h. CM was filtered through a 0.2 µM filter and stored at -20°C until ready for use or analysis. Luminex® xMAP technology was used to quantify SASP factors in CM. Multiplexing analysis was performed using the Luminex 100 system (Luminex) by Eve Technologies using human multiplex kits (Millipore).

PKH26 cell labelling

Human cycling-CPCs, doxorubicin (Dox)-induced Senescent-CPCs or c-kit^{neg} cells ($5x10^5$) were harvested and washed in serum-free DMEM/F12 medium before being resuspended in 25µl diluent C (Sigma-Aldrich). An equal volume of PKH26 labelling solution (4µM; Sigma-Aldrich) was added to the cell suspension and mixed thoroughly. Cells were incubated for 2 minutes at room temperature in the dark, before adding an equal volume of DMEM/F12 + 10% FCS to halt the labelling reaction. Cells were then centrifuged and washed three times in PBS before transferring to a new Eppendorf tube and re-suspending in 15µl PBS for transplantation *in vivo*. Initial PKH26 labelling was validated using flow cytometry compared to unlabelled cells. PKH26 labelled cycling CPCs and c-kit^{neg} cells were also propagated *in vitro* to monitor label retention over culture passage (P1-P9). To quantify PKH26^{pos} labelling, cells were cytospun and counterstained with DAPI. The total number of positive cells per total nuclei were quantified from a minimum of 5 random fields at x20 magnification.

Acute myocardial infarction model

The following experimental procedures were conducted in accordance with the regulations for animal testing, directed by the Home Office and stipulated under the Animals (Scientific Procedures) Act 1986. Non-obese diabetic (NOD), severe combined immunodeficiency (SCID) IL2Rgamma-c null mice [NSG; Charles River, UK] were used for all acute myocardial infarction studies. Mice were group housed in conditions of 22±1°C room temperature and 50±1% relative humidity on a 12-hour light-dark cycle. Standard laboratory mice chow and water were available *ad libitum*. Mice were 8-10 weeks old and were randomly assigned to each treatment group. Sample sizes were estimated according to power calculations performed on Minitab Software. Sham (n=5), PBS (n=4), Cycling-CPCs (n=7), Sen-CPCs (n=5), c-kit^{neg} cells (n=6)

Prior to echocardiography, mice were weighed and anaesthetised in an anaesthetic chamber at 0.5ml/min O_2 and 4% Isoflurane. The depth of anaesthesia was assessed by a change in the

breathing pattern of the mouse and the absence of the pinch withdrawal reflex. Once under deep anaesthesia, mice were placed onto an imaging station (Vevo, Netherlands). Anaesthesia was regulated to maintain heart rate between 400-500 beats per minute monitored by ECG recordings, and body temperature was maintained at 37 ± 0.5°C using a temperature probe. Mice were depilated and ultrasound gel was applied onto the vicinity of the chest (Aquasonic 100, Germany). Parasternal long and short axis images of the heart were taken using the Vevo 770 imaging system (Netherlands) and measurements taken using the B-mode and M-mode features. Ultrasounds were taken prior to left anterior descending coronary artery ligation (Baseline) and 1 and 4 weeks post-surgery. The following measurements were taken: interventricular septum thickness at end-systole/diastole ($IVS_{s/d}$), left ventricular end diastolic volume/ left ventricular end systolic volume (LVEDV/LVESV), and left ventricular anterior wall thickness at end-systole/diastole ($LVAW_{s/d}$). Ejection Fraction (EF), Stroke Volume (SV), and Fractional Shortening (FS) were measured manually using the left ventricular interventricular dimension (LVID) Trace tool and were also derived by long and short axis calculations.

Left anterior descending (LAD) coronary artery ligation was achieved as follows: once under deep anaesthesia, mice were intubated and attached to a mechanical ventilator (200ml stroke volume; 150 breaths per minute, Hugo Sachs Electronik, Germany), anaesthesia was reduced to 2% isoflurane at 0.5ml/min for the duration of the surgical procedure. Proceeding depilation, the thoracic area was sterilised and a midline incision along the skin overlying the sternum was performed to expose the muscle. A left-sided thoracotomy between the fourth and fifth rib was performed to expose the heart. Following the separation of the pericardial sac, the coronary artery was ligated using 6-0 silk suture (Ethicon, US) using the left atrium and aorta as entry and exit landmarks, respectively. The force of the suture was determined by the appearance of a color change in the left ventricle. Following the permanent ligation, the heart was assessed for atrial fibrillation for a 10 minute interval and was preceded by the closure of the thorax and the skin using 5-0 silk suture (Ethicon, US). In sham-treated mice, a permanent ligature was not tied around the LAD coronary artery. Immediately following MI, mice were injected intramyocardial with PKH26 labelled cycling-CPCs, Senescent-CPCs, or c-kit^{neg} cells (all $5x10^5$) delivered in a total volume of 15µl PBS, delivered across two sites at the border zone. Directly following MI+cell injection, mice were implanted with an osmotic pump (Alzet®, USA) loaded with a 0.2M solution of BrdU (MP Biomedicals), releasing the

thymidine analogue for 14 days. Anaesthesia was then removed and mice were taken off mechanical ventilation after observing an independent breathing pattern dyssynchronous to that of the ventilator. Methadone analgesic was administered, Comfortan® (1mg/kg; Dechra) i.m., and mice were kept in heat boxes overnight in a constant warm environment (27±1°C and a relative humidity of 50±5%). 24 and 48 hours following surgery, body weight and signs of pain were checked and post analgesic was administered as and when necessary. Following surgery, mice were housed singly caged with access to water and chow *ad libitum.* Echocardiography measurements were taken at baseline (BL), 7 days, and 28 days post-MI, after which mice were sacrificed.

Senolytic drug treatment, viability, TUNEL staining *in vitro*

Cycling-competent human CPCs and Senescent-CPCs, induced by Doxorubicin (as described above), were plated in separate 24-well plates. Senescence was confirmed by SA-β-gal staining and the percentage of SA-β-gal^{pos} cells at day 0 was over 80% prior to senolytic drug treatment. After 3 days of either Dasatinib (LC Laboratories), Quercetin (Sigma-Aldrich,ST. Louse, MO), Navitoclax (Selleckchem, Houston, TX), Fisetin (Sigma-Aldrich, St. Louise, MO) at doses 0.5µM to 20µM or vehicle exposure, the number of Senescent-CPCs was quantified by SA-β-gal staining.

Cell viability was measured by crystal violet (CV) staining at day 0 then following 3 days of drug exposure. Briefly, cells were washed twice with PBS, fixed in methanol on ice for 10 minutes, and stained with 0.5% crystal violet (Sigma-Aldrich) in methanol for 15 minutes at room temperature. Cells were washed with deionized water and staining intensity was measured using a light microscope, with a minimum of 5 random fields of view at 4x magnification taken per well. The captured images were analyzed using ImageJ software (Fiji, USA).

Cell apoptosis was detected using the terminal deoxynucleotidyl transferase (TdT)-mediated dNTP nick end labelling (TUNEL) assay (Trevigen) according to the manufacturer's instructions. Cells were cultured in 24-well plates as described previously and then treated with either vehicle or D+Q for 16 hours. To perform TUNEL staining, cells were incubated with proteinase K for 15 min at room temperature then a labelling buffer containing TdT enzyme, TdT dNTP, and Co^{2+} was applied for 1h at 37°C. Positive controls were incubated with TACS nuclease, while negative controls omitted the TdT enzyme from the labelling

mix. After labelling, cells were washed in stop buffer for 5 min and were then incubated with Strep-Fluorescein in 0.05% Tween:PBS for 20 min at room temperature. After washing, cells were counterstained with DAPI (Sigma-Aldrich) and mounted using Vectasheild mounting media (Dako). The cells were viewed and imaged using an ApoTome fluorescent microscope (Zeiss). A minimum of 5 random fields of view at x20 magnification were analysed and number of TdT-positive cells expressed as a percent of total nuclei.

Co-culture assays

CPCs were plated in the bottom well of 0.4μm pore Costar Transwell 24-well plates (Corning) and, once attached, were induced to senescence with Doxorubicin as described above and maintained for 30 days. Cycling-competent CPCs were plated at low density (200 cells/well) onto the Transwell membrane 1 day prior to co-culture. Cycling-competent CPCs were then combined with either senescent CPCs or cultured alone (control wells) and maintained for 7 days. At this time point, cultures were either fixed for analysis, conditioned media were collected, or senolytics were applied to transwells for 3 days to selectively clear senescent cells (as described above). After senolytic clearance, a sub-set of wells were fixed and stained for SA-β-gal (as described previously) to confirm effective clearance. Cycling-competent CPCs were maintained for a further 7 days, at which point conditioned media were collected for luminex analysis (as described above) and cultures fixed for analysis.

INK-ATTAC mice and drug treatments

The following experimental procedures were conducted in accordance with Mayo Clinic Institutional Animal Care and Use Committee (IACUC) guidelines. Both stocks of *INK-ATTAC* and C57BL/6 wild type mice were bred and aged at Mayo Clinic. The generation and characterization of *INK-ATTAC* mice has been described previously (Baker, Wijshake et al. 2011, Xu, Palmer et al. 2015). Mice were housed in ventilated cages and maintained in a pathogen-free, accredited facility under a 12 h light–dark cycle with constant temperature (23°C) and access to food (standard mouse diet, Lab Diet 5053, St. Louis, MO) and water *ad libitum*. 24-32 month old mice were randomly assigned to treatment groups and injected intraperitoneally (i.p.) with either vehicle (60% Phosal-50PG, 30% PEG-400, and 10% ethanol) or AP20187 (B/B homodimerizer, Clontech, 10 mg AP20187/kg body mass) or administered by oral gavage vehicle (66% Phosal-50PG, 10% ethanol, and 30% PEG-400) or senolytic drugs (5mg/kg Dasatinib + 50mg/kg Quercetin diluted in vehicle) for 3 consecutive days every 2 weeks for 2 months as shown in Fig 6a.

This dosing regime was chosen because it has been shown to be effective at clearing senescent cells in chronologically aged *INK-ATTAC* mice in previous studies (Xu, Palmer et al. 2015, Roos, Zhang et al. 2016). Another group of *INK-ATTAC* mice aged 3 months (n=10) and 22 months (n=10) were randomly assigned to treatment groups and injected intraperitoneally (i.p.) with either vehicle or AP20187 for 2 consecutive days every 2 weeks for 2 months as shown in Supplementary Fig 10c. These mice were injected (i.p) with EdU (123 $mgkg^{-1}$) 2 hours prior to sacrifice.

Tissue collection, immunohistochemistry, and confocal imaging

Mice were sacrificed at either 4 days or 28 days after MI with the hearts arrested in diastole with cadmium chloride solution (Sigma), removed, and embedded in OCT compound (Tissue-Tek) at ⁻80°C before preparing 10µm sections. To identify transplanted cells, transverse sections were identified by PKH26 fluorescent labelling (Sigma-Aldrich) and nuclei counterstained with DAPI. Sections were assessed for co-localisation of PKH26 labelling and myocytes or vascular structures using antibodies against α-sarcomeric actin (cardiomyocytes; 1:250 dilution, 1hr at 37°C) or vWF (endothelial cells; 1:250 dilution, 1 hr at 37°C) respectively. Images were taken using a confocal microscope (Nikon A1) at x63 magnification. To quantify PKH26-cell engraftment, the total number of $PKH26^{pos}$ cells per total nuclei were quantified from a minimum of 5 random fields at x20 magnification. To measure scar size, sections from the apex and mid-region of the heart were stained with Haematoxylin Van Gieson (HVG, Fisher Scientific, UK) using standard procedures. Fibrosis was visualised using a light microscope (Zeiss, Axioscope MTB2004) connected to Axiovision software (Zeiss) where sections were captured at x5 magnification and stitched to reconstruct an image of a section of the whole heart. Analysis was performed using Image J software (NIH). Data were acquired by measuring the area of fibrosis (red) across the apex and mid-regions of the heart using an average of 5 images per heart and the area of left ventricle and were expressed as a percentage Mean ± SEM. (Zeiss Apotome fluorescent microscope, Oberkochen, Germany). Proliferating cells were identified using antibodies against BrdU (Roche) (Supplementary Table 1) and nuclei counterstained with DAPI. A minimum of 5 random fields of view at x20 magnification were analysed and data expressed as percent of BrdU-positive cells per total nuclei. Sections were also assessed for number of newly formed myocytes using antibodies against BrdU (Roche) and α-sarcomeric actin (1:250 dilution, 1hr at 37°C). A minimum of 5 random fields of view were taken at the border zone using at x63 magnification per animal. The number of BrdU-positive cardiomyocytes

were expressed as a percent of total cardiomyocyte nuclei. Newly formed vascular structures were detected by staining for BrdU (Roche) and vWF (1:250 dilution, 1hr at 37°C). A minimum of 5 random fields of view at x20 magnification were analysed at the border zone and number of BrdU-positive capillaries (1-2 nuclei) expressed as a percent of total capillaries. Quantification of PKH26 and α-sarcomeric actin of vWF localisation were used to determine in vivo differentiation of donor CPCs. A minimum of 5 random fields of view (FOV) at 20x magnification at the border zone were analysed and mean number of PKH26-positive α-sarcomeric actin- or vWF-positive cells expressed per FOV. In all cases sections were imaged and analysed using an A1 confocal microscope (Nikon). All antibodies used in immunostaining are listed in **Supplementary Table 2**.

INK-ATTAC and wild-type C57BL/6 mice were sacrificed 4 days after the last dose of the last course of treatment with AP20187 or senolytic drugs. Hearts were explanted and cut into two parts. One half was fixed in 4% formaldehyde for 24 hrs and embedded in paraffin. The other half was snap-frozen in liquid nitrogen for subsequent RT–qPCR analyses as described below. 10µm transverse heart sections were cut on a microtome (Leica) and mounted onto microscope slides. Antigen retrieval was achieved using Target Retrieval Solution, Citrate pH 6 (DAKO). Sections were stained for c-kit (1:500 dilution, overnight at 4°C), Sca-1 (1:500 dilution, overnight at 4°C), CD45 (1:1000 dilution, 1 hr at 37°C), CD34 (1:1000 dilution, 1 hr at 37°C), CD31 (1:1000 dilution, 1 hr at 37°C), Ki67 (1:500 dilution, 1 hr at 37°C) and α-sarcomeric actin (1:100 dilution, 1 hr at 37°C). Secondary antibodies were Alexa Fluor 488, 594 or 633 (Life Technologies). The nuclei were counterstained with DAPI) (Sigma-Aldrich). Sections were mounted in Vectashield (Vector labs) and analysed and scanned using confocal microscopy (A1 confocal, Nikon). CPCs were identified as being Sca-1- and c-kit-positive, and CD45-, CD34-, CD31-negative. Proliferating CPCs were identified as Sca-1- and c-kit-positive and Ki67-positive. Proliferating cardiomyocytes were identified as Ki67-positive or EdU-positive and α-sarcomeric actin positive. CPCs were counted in a minimum of 10 random fields of view at x40 magnification and the number of CPCs expressed as a percent of total nuclei. Proliferating cardiomyocytes were counted across random fields of view of 3 cross sections at x63 magnification. A total of 1500 cardiomyocytes were counted per animal per section and the number that were Ki67- or EdU-positive expressed as percentage of cardiomyocytes. Cardiomyocyte diameters were measured across the nucleus on transverse sections of α-sarcomeric actin-positive stained cardiomyocytes using ImageJ software. A minimum of 60 cardiomyocytes were measured

per animal. LV fibrosis was detected using HVG staining as described above. All antibodies used in immunostaining are listed in **Supplementary Table 2**.

Real-time quantitative polymerase chain reaction (RT–qPCR)

To measure human gene expression, total cellular RNA was extracted and reverse transcription was performed as described previously (Lewis et al. 2014). RT-qPCR reactions were run with SYBR Green PCR Master Mix (Bio-Rad) and primers (IDT) (**Supplementary Table 3**). Human *GAPDH*, *B-actin*, and *TBP* were used as reference genes. qPCR conditions were as follows: 95°C for 5 min, 40 × (95°C for 15s, 60°C for 30s, and 72°C for 30s). All reactions were run in triplicate on a CFX-connect Real-Time PCR System (Bio-Rad) and analyzed using Graphpad software.

From mouse hearts, total RNA was extracted using Trizol (Thermo Fisher Scientific) and reverse transcription was performed using an M-MLV reverse transcriptase kit as described previously (Xu et al. 2015, PNAS) To measure total $p16^{Ink4a}$ expression, RT-qPCR reactions were performed with TaqMan (Applied Biosystems, Carlsbad, CA) Fast Advanced Master Mix and TaqMan primer- probe gene assay (Thermo Fisher Scientific). Mouse TATA-binding protein (*TBP)* was used as a reference gene and all reactions were run in triplicate on an ABI Prism 7500 fast Real Time System (Applied Biosystems, Carlsbad, CA) and analyzed using Graphpad software.

Statistical analysis

Data are reported as mean ± SEM or mean ± SD. The data displayed normal variance. The experiments were not randomized, except for the *in vivo* animal studies as described above. The investigators were blinded to allocation during experiments and outcome assessment. Significance between 2 groups was determined by Student's *t*-test and in multiple comparisons by the analysis of variance (ANOVA) using GraphPad Prism (GraphPad Software). In the event that ANOVA justified *post-hoc* comparisons between group means, these were conducted using Tukey's multiple-comparisons test. P<0.05 was considered significant.

Supplementary Figure 1

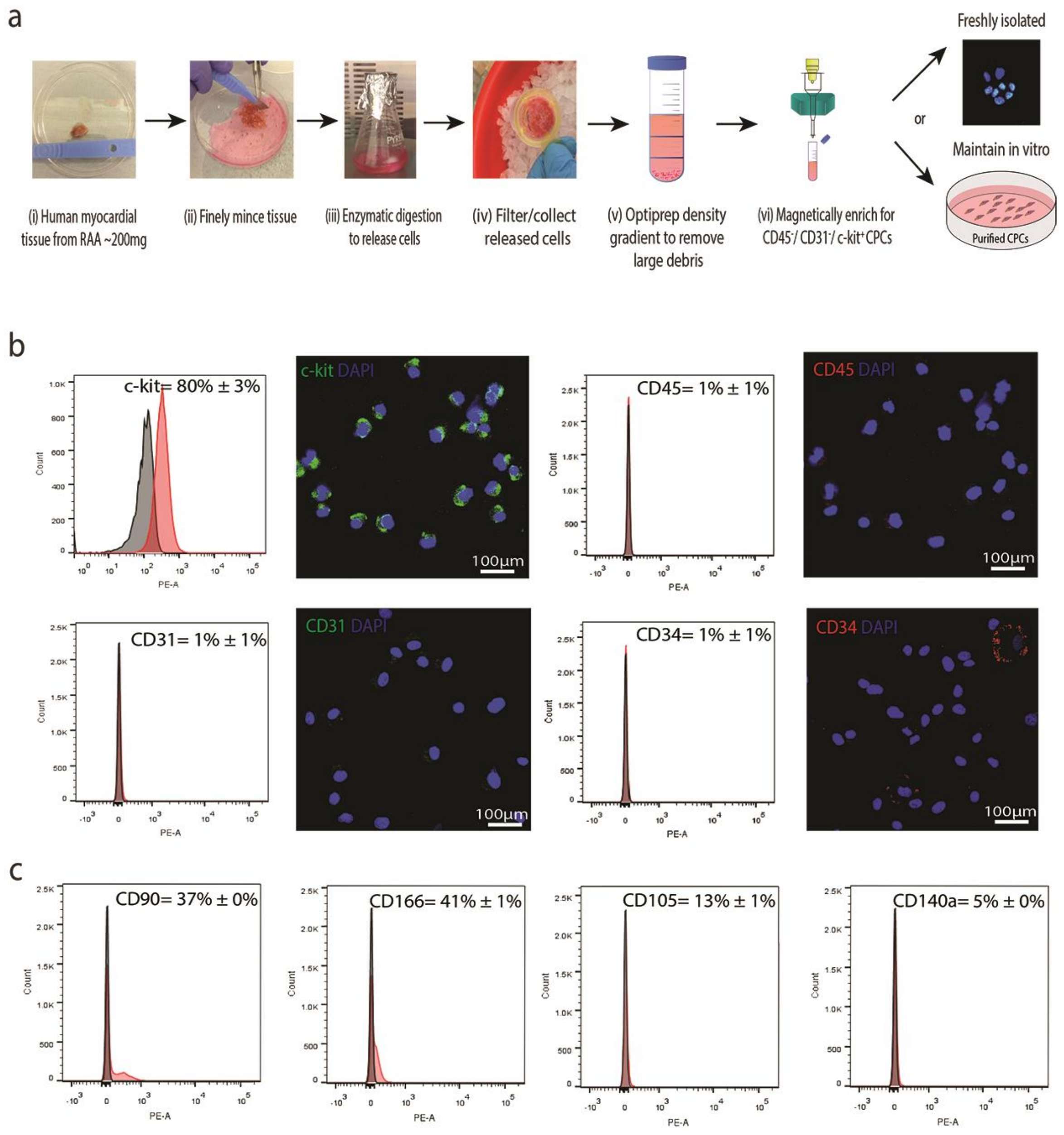

d

Parameter		c-kit^{pos} CD45^{neg} CPCs/gram of tissue	N	*p value*
**Total**		45,477 ± 30,081	40	*n/a*
**Age <70 years**		40,750 ± 27,093	19	*p>0.05*
**Age >70 years**		48,853 ± 32,469	21	
**Male gender**		47,116 ± 23,211	21	*p>0.05*
**Female gender**		29,246 ± 10,740	17	
**Pathology**	*Valve Disease*	32,400 ± 12,043	7	*p>0.05*
	Coronary Disease	44,953 ± 26,855	18	
	Aortic Disease	49,883 ± 34,427	20	

Supplementary Figure 1. Isolation of CPCs from the human heart. (a) Human myocardial tissue is obtained from the right atrial appendage (RAA) of consenting donors. Tissue is finely minced and then sequentially digested using collagenase II for 3 min intervals at 37°C with agitation for a total duration of ~30 min. The quenched cell suspension is filtered and an Opti-Prep density gradient performed to remove large debris and isolate the

small cell population. In order to purify CPCs from the small cell population, MACS depletion of CD31^{pos}/CD45^{pos} cells followed by c-kit^{pos} sorting is performed. (b) Representative flow cytometric and immunostaining analysis of freshly isolated c-kit^{pos}/CD31^{neg}/CD45^{neg}/CD34^{neg} CPCs. (c) Flow cytometric profiling of freshly isolated CPCs for CD90, CD166, CD105 and CD140a, n=2 subjects. (d) Quantification of the total yield of c-kit^{pos}/CD45^{neg} CPCs harvested from myocardial tissue samples grouped by age, gender and pathology.

Supplementary Figure 2

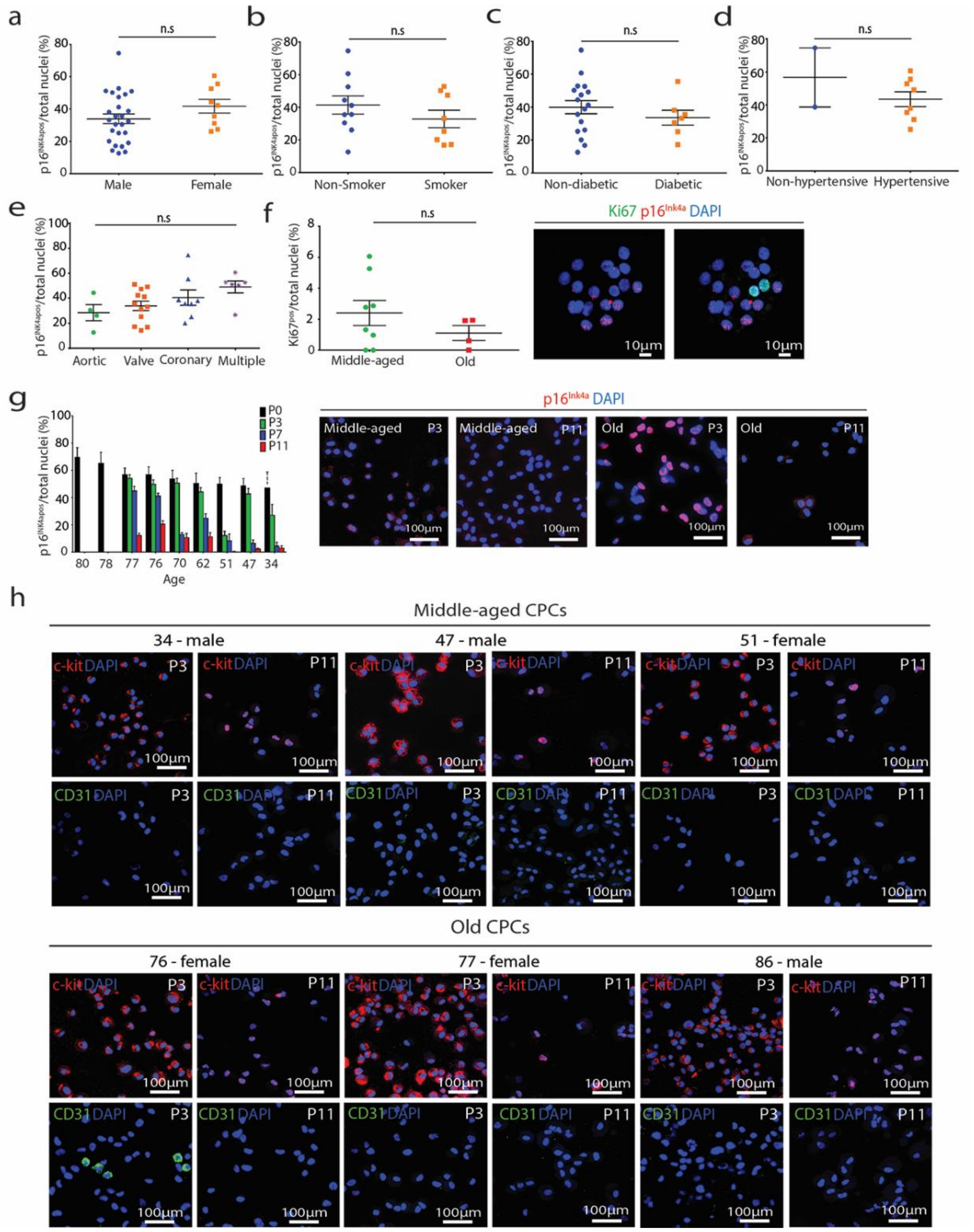

Supplementary Figure 2. Freshly isolated p16$^{INK4A\text{-}pos}$ CPCs are withdrawn from the cell cycle. (a) Quantification of freshly isolated CPC p16^{Ink4a} expression, each data point represents the mean number of p16$^{INK4A\text{-}pos}$ cells per total nuclei, calculated from 20 fields of view for each individual donor. Data are grouped by (a) gender (n=27 male, n=8 female), (b) smoking (n=10 non-smoker, n=8 smokers), (c) diabetes (n=17 non-diabetic, n=7 diabetic), (d) hypertension (n=2 non-hypertensive, n=8 hypertensive), (e) pathology (n=4 aortic, n=12 valve, n=8 coronary, n=6 multiple). (c) Quantification of freshly isolated CPC Ki67 expression, each data point represents the mean number of Ki67^{pos} cells per total nuclei, calculated from 20 fields of view for each individual donor (n=8 middle-aged, n=4 old). Representative immunofluorescent image showing mutually exclusive expression of Ki67

(green) and p16^{Ink4a} (red) in freshly isolated CPCs. (d) Quantification of CPC p16^{INK4A} expression *in vitro*, each bar represents the mean number of p16$^{INK4A\text{-}pos}$ cells per total nuclei at P0, P3, P7 and P11 for each individual donor. (e) Representative immunofluorescent images of CPCs from middle-aged and old donors at early passage (P3) and late passage (P11) showing expression of the senescence marker, p16^{INK4A} (red). (c) CPCs isolated from middle-aged and old donor hearts were characterised for c-kit (red) and CD31 (green) at early (P3) and late (P11) passage.

Supplementary Figure 3

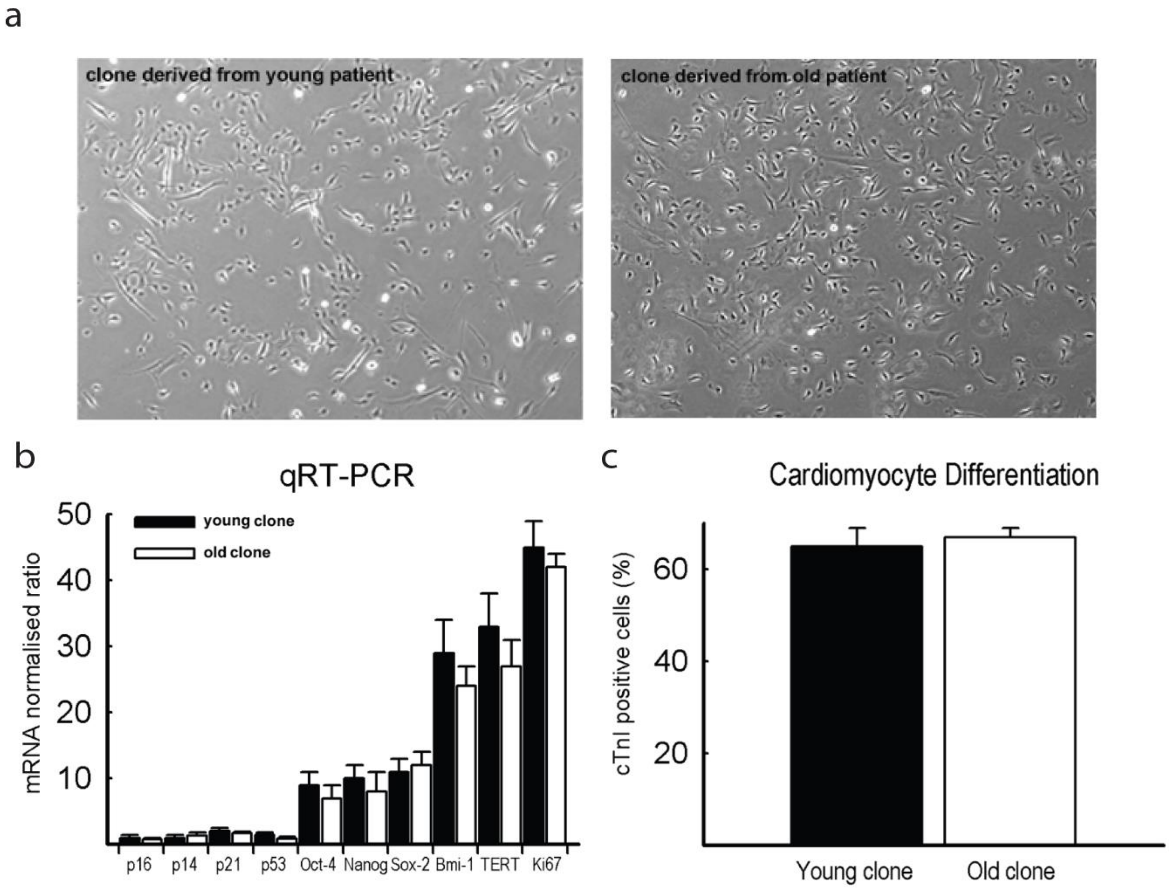

Supplementary Figure 3. Single cell-derived CPC clones from young and old hearts are indistinguishable. (a) Representative micrographs of single cell-derived clones from young (22, 30 and 33 years; left) and old (74, 75, 83 years; right) donor hearts. (b) Relative gene expression of pluripotency, cell cycle and senescence transcripts relative to internal reference gene. (c) $cTnI^{-pos}$ cells per total nuclei calculated from 10 fields of view for each clone. Data are Mean ± SD for n=3 subjects.

Supplementary Figure 4

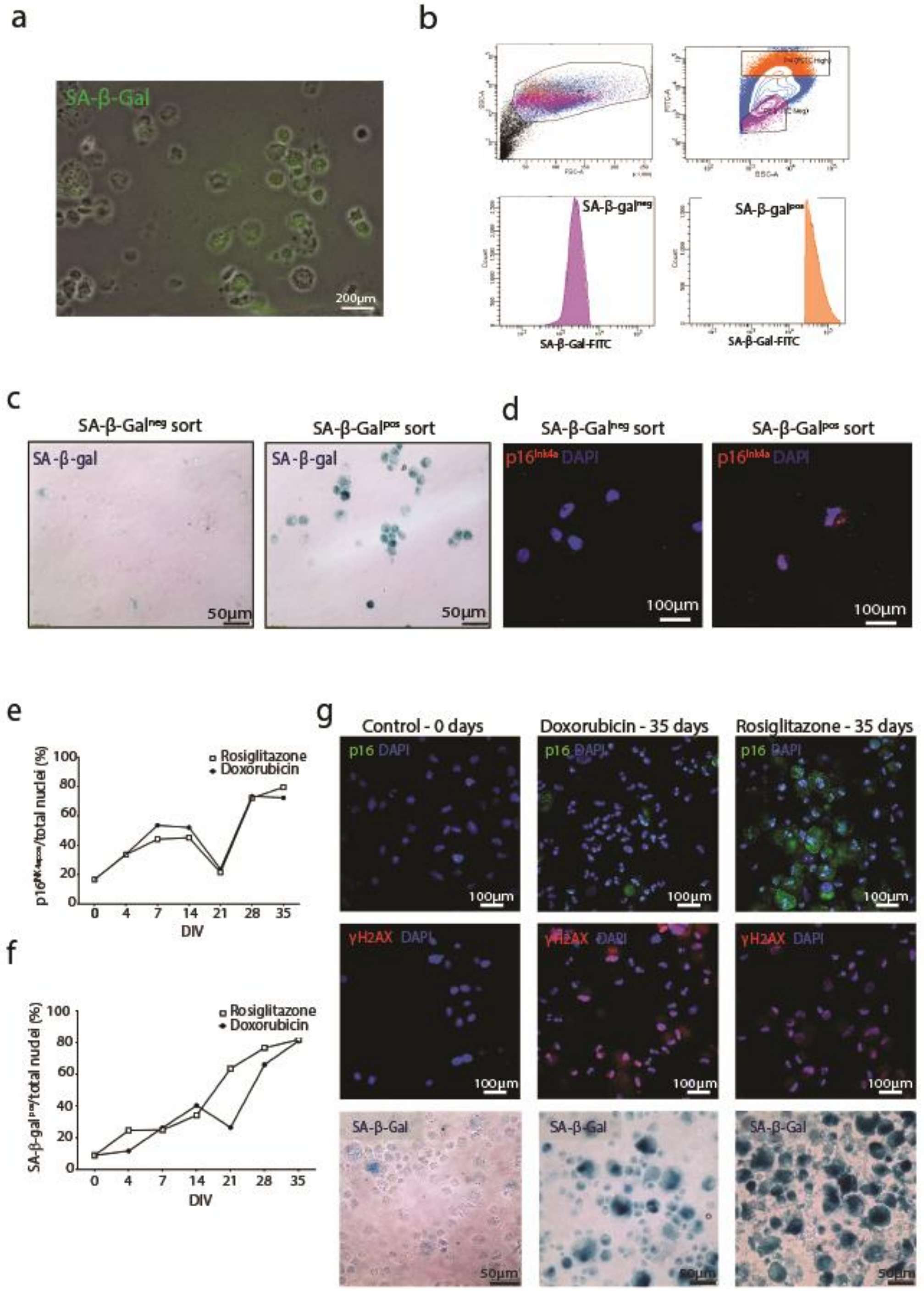

Supplementary Figure 4. Expression of senescence markers in senescence-induced CPCs. (a) Representative micrographs of freshly isolated C_{12}FDG SA-β-gal^{pos} sorted CPCs (green). (b) Dot plot and histograms showing FACS gating strategy. FACS sorted CPCs were cytospun and validated using (c) SA-β-gal staining (blue) and (d) p16^{INK4A} immunostaining (red). Line graphs represent mean percentage of (e) p16$^{INK4A\text{-}pos}$ and (f) SA-β-gal^{pos} cells per total nuclei for Rosiglitazone and Doxorubicin-treated CPCs for time points 4, 7, 14, 21, 28 and 35 days post-treatment. (g) Representative micrographs showing control untreated CPCs (left), Doxorubicin-treated CPCs (middle) and Rosiglitazone-treated CPCs (right) stained for p16^{INK4A} (green), γH2AX (red) or SA-β-gal (blue).

Supplementary Figure 5

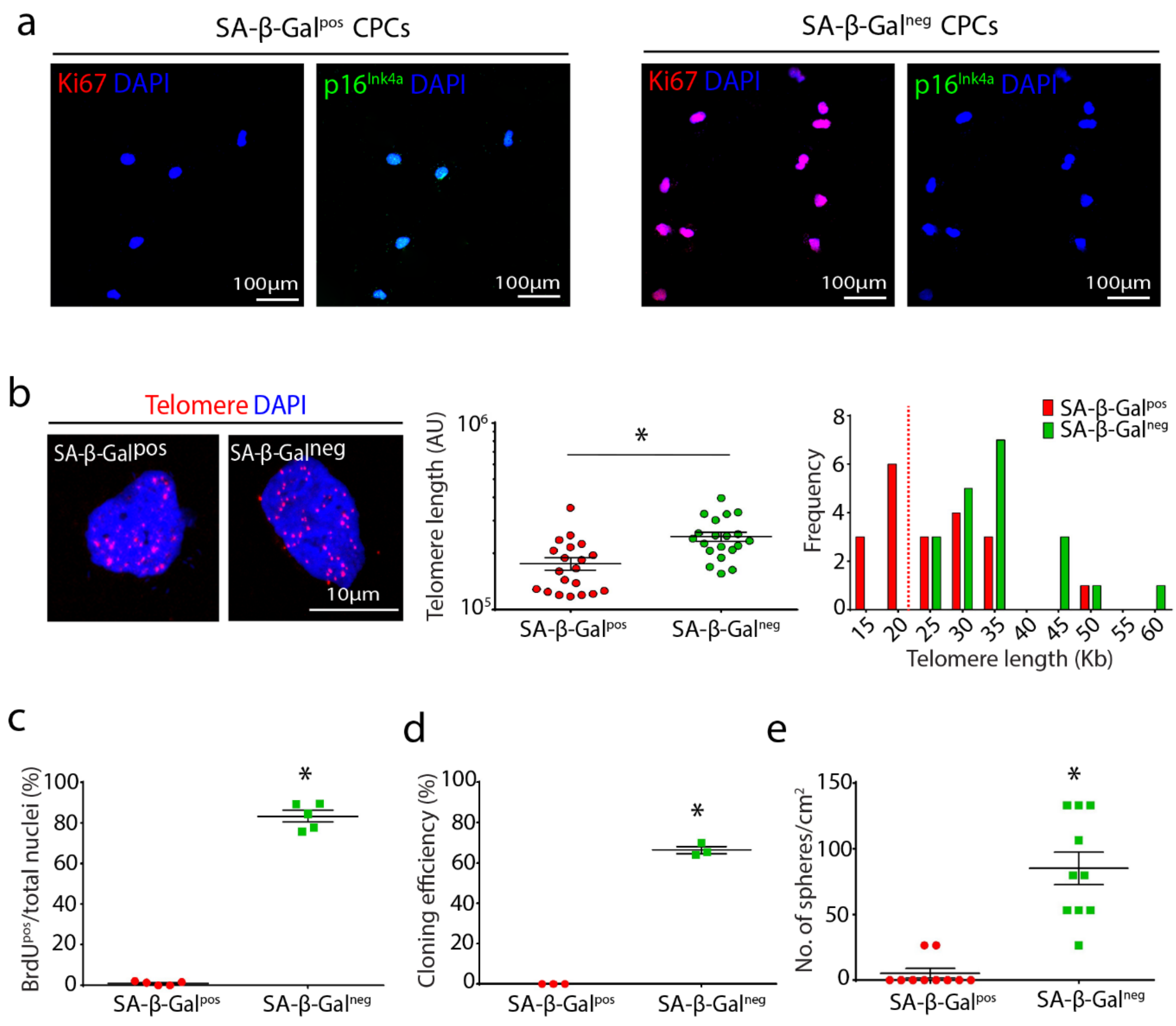

Supplementary Figure 5. Characterisation of sorted SA-β-gal^{pos} vs. SA-β-gal^{neg} CPCs. (a) Representative immunofluorescent images of SA-β-gal^{pos} and SA-β-gal^{neg} CPCs stained for Ki67 and p16^{Ink4a}. (b) Representative immunofluorescence images and quantification of SA-β-gal^{pos} and SA-β-gal^{neg} CPCs Q-FISH telomere staining, each data point represents the telomere signal intensity (red) per total nuclei (blue) calculated from 20 cells each (**P*=0.0009). Mouse cell lines with known telomere length, L5178Y-S (10 Kb) and L5178Y-R (79 Kb) were used to determine telomere length histograms calculated from 20 cells each. (c) Proliferative capacity was determined using a BrdU incorporation assay, each data point represents the mean number of BrdU^{pos} cells per total nuclei. (d) Cloning efficiency was determined as the percentage of single cells that formed colonies after 7 days, each data point represents the mean cloning efficiency. (e) Quantification of the mean number of spheres per cm^2. In each scatter graph lines represent mean ± SEM, **P*<0.0001 determined using a Student’s t-test.

Supplementary Figure 6

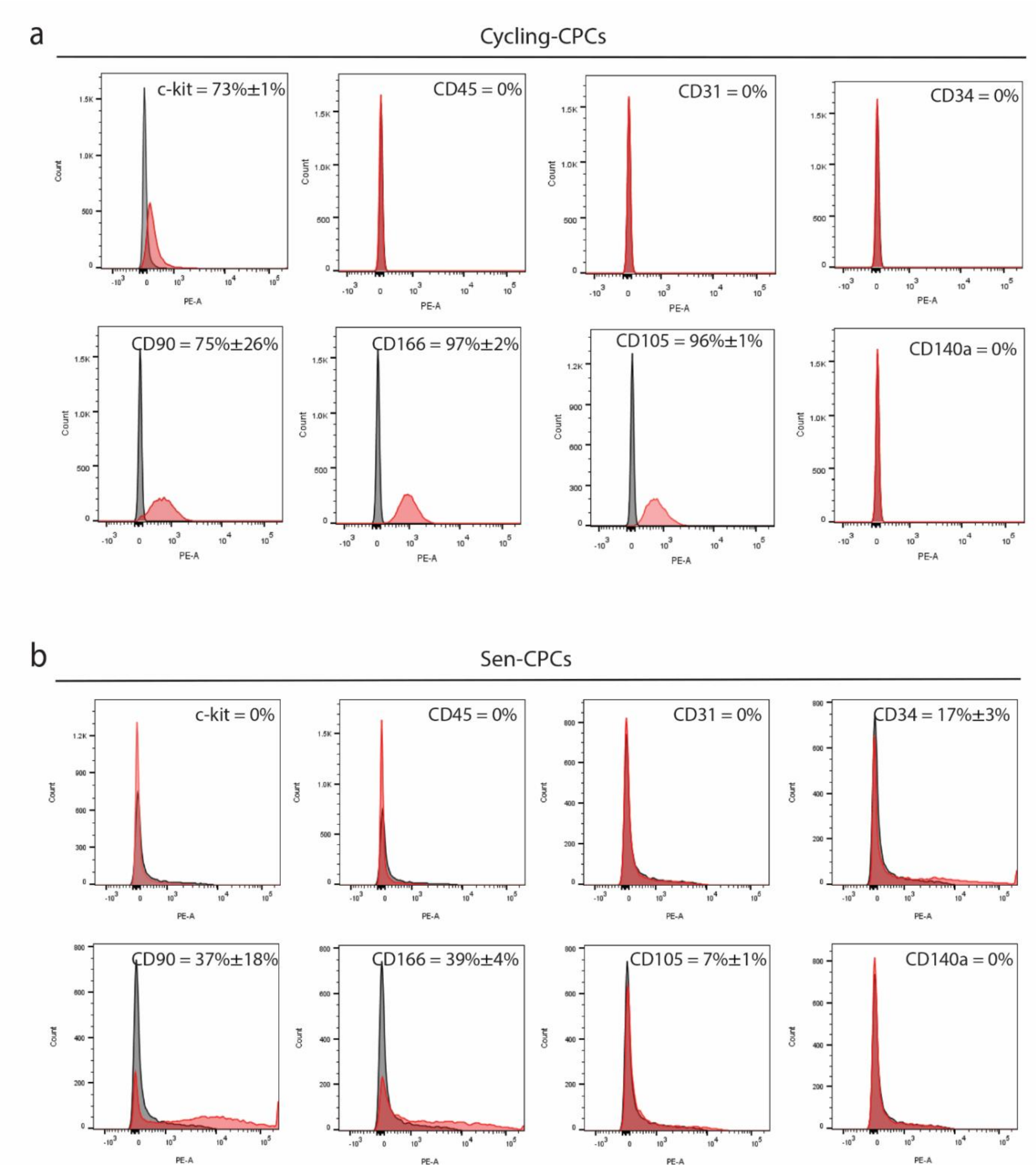

Supplementary Figure 6. Phenotype of CPCs transplanted *in vivo*. Flow cytometric analysis of CPCs. Representative histograms show the percentage of total cells expressing target antigens (red) against isotype control (grey) for (a) cycling-CPCs P6 (n=2) and (b) Dox-induced Sen-CPCs P6, n=2 subjects.

Supplementary Figure 7

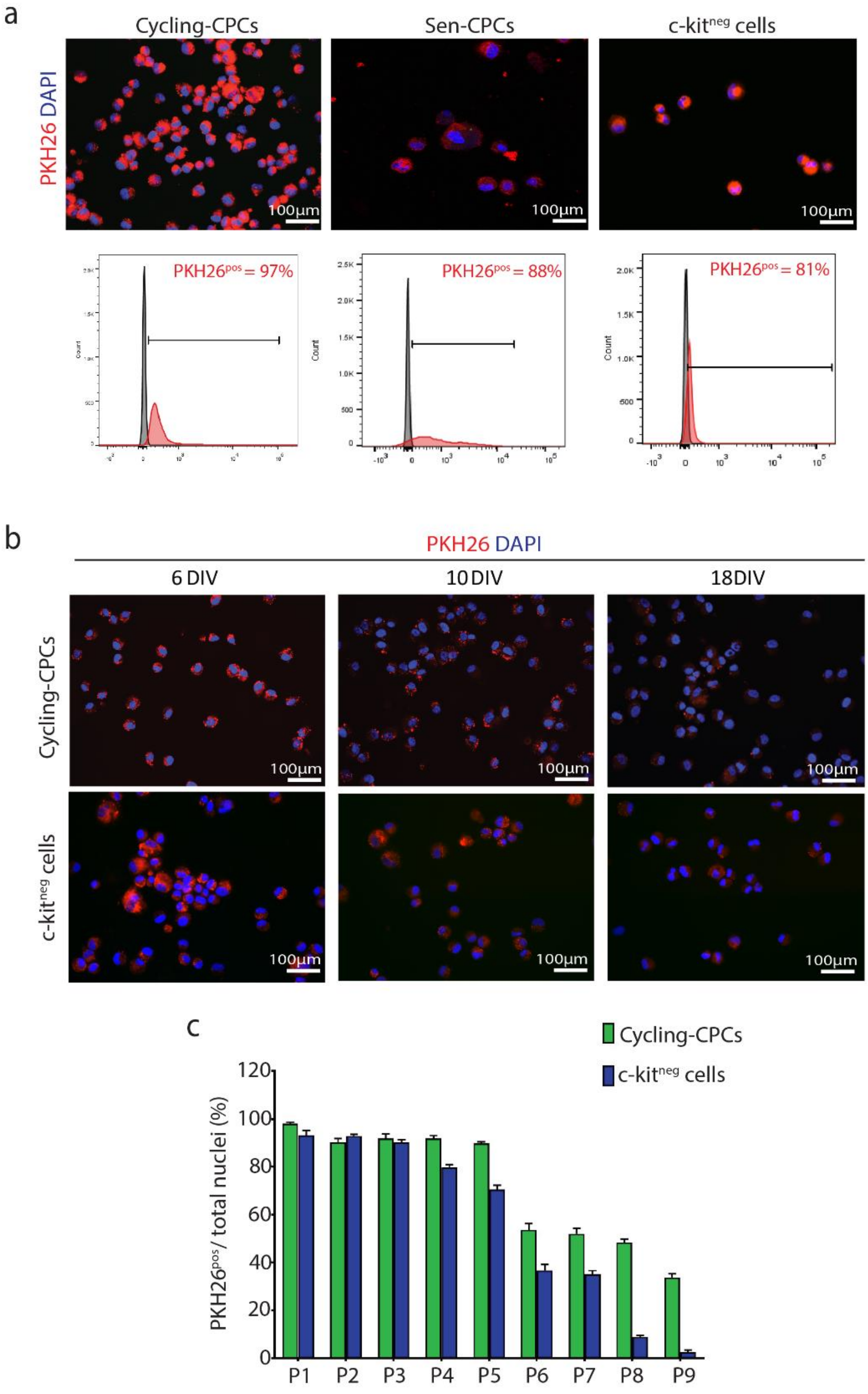

Supplementary Figure 7. PKH26 labelling of CPCs for tracking *in vivo.* (a) Representative immunofluorescent images and quantification of PKH26 labelled cells (red) against unlabelled control (grey histogram). (b) Representative immunofluorescent images of PKH26 labelled cells (red) propagated *in vitro*, cytospun and analysed at 6, 10 and 18 DIV. (c) Quantification of CPC PKH26^{pos} label retention *in vitro*, each bar represents the mean number of PKH26^{pos} cells per total nuclei at P0-P8 for cycling-CPCs and c-kit^{neg} cells calculated from 5 fields of view each.

Supplementary Figure 8

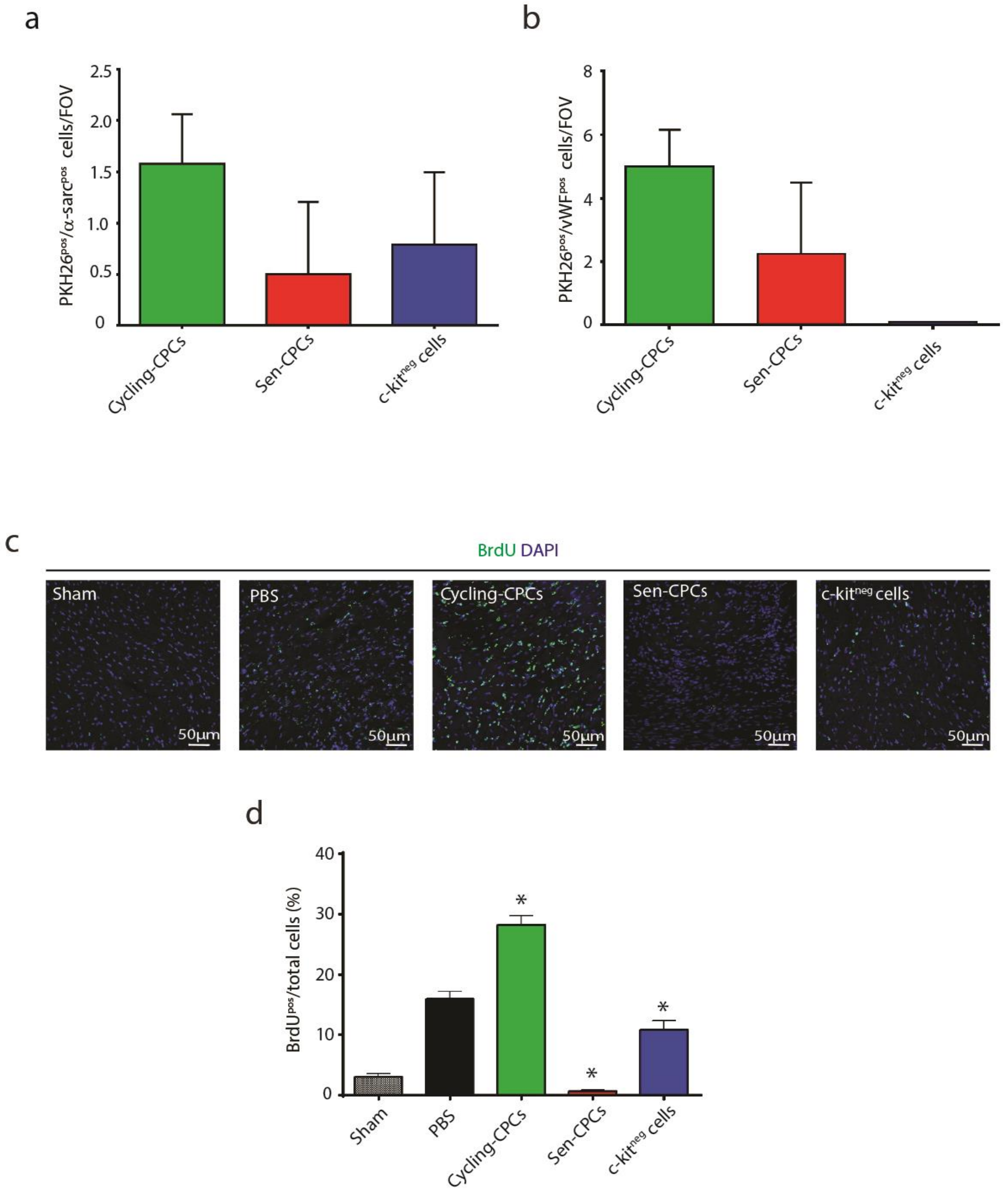

Supplementary Figure 8. Total BrdU proliferation and co-expression of PKH26 and differentiation marker expression *in vivo*. (a) Quantification of PKH26 and α-sarcomeric actin co-expression per field of view (n=2-3 mice per group). (b) Quantification of PKH26 and vWF co-expression per field of view (n=2-3 mice per group). (c) Representative immunofluorescent images of BrdU labelled (green) myocardial tissue. (d) Quantification of BrdU^{pos} cells per total nuclei (*P<0.0001 vs. PBS, n=5 mice per group). All data represent mean ± SEM. Statistical analysis was performed using one-way ANOVA with Tukey's multiple comparison test.

Supplementary Figure 9

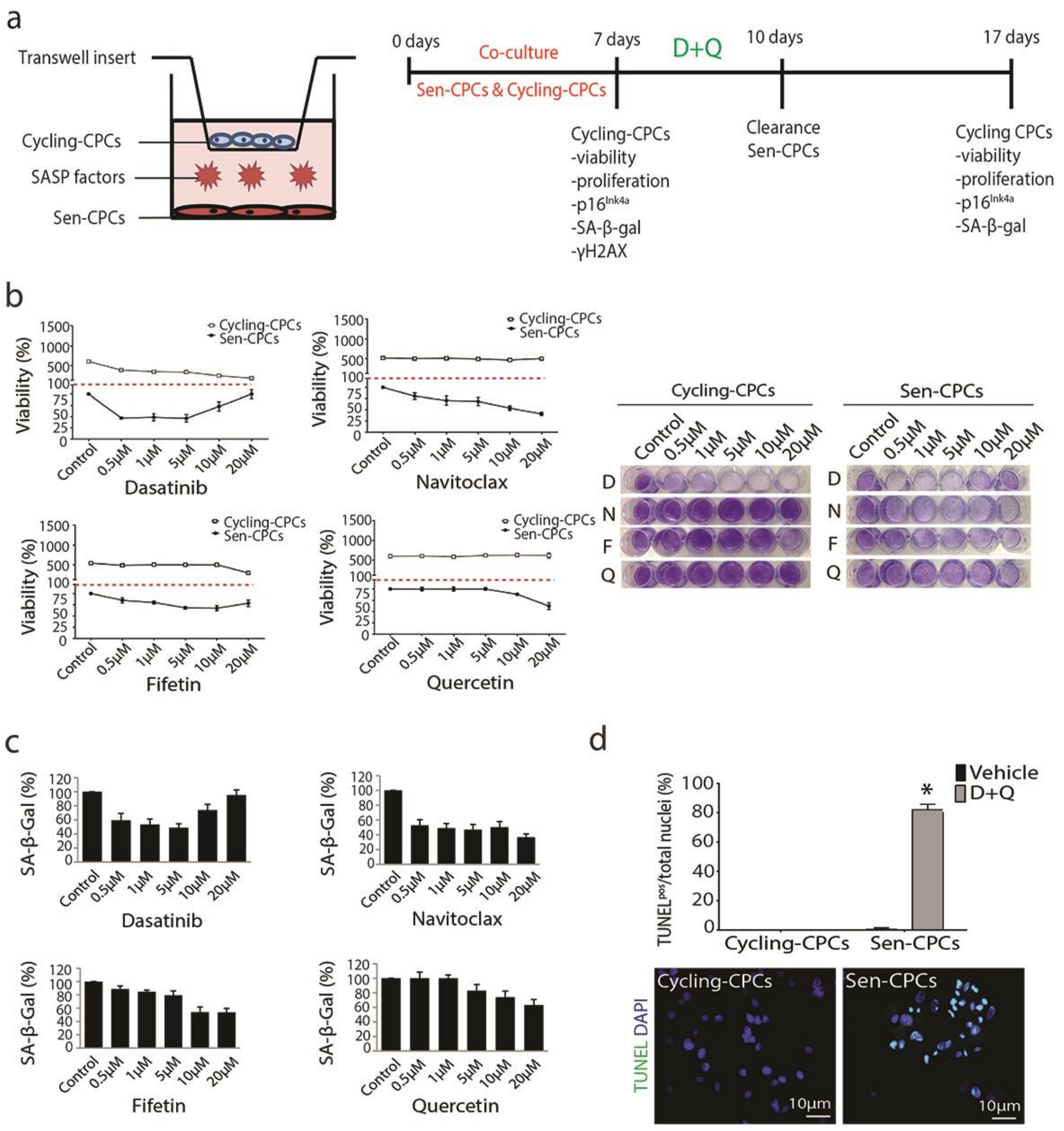

Supplementary Figure 9. Elimination of senescent-CPCs using senolytic drugs. (a) Experimental design utilised in transwell co-culture senolytic clearance experiments. (b) Viability of human CPCs exposed to different senolytic drugs for 3 days. The red line denotes cell viability on day 0 prior to senolytic exposure. (c) Dox-induced Sen-CPCs were exposed to different senolytics for 3 days and clearance was quantified using SA-β-gal staining relative to untreated control. (d) D+Q exposure resulted in an increased number of apoptotic cells in Sen-CPC cultures after 16h. All data are Means±SEM, n=2 independent experiments $^*P<0.0001$ by two-way ANOVA.

Supplementary Figure 10

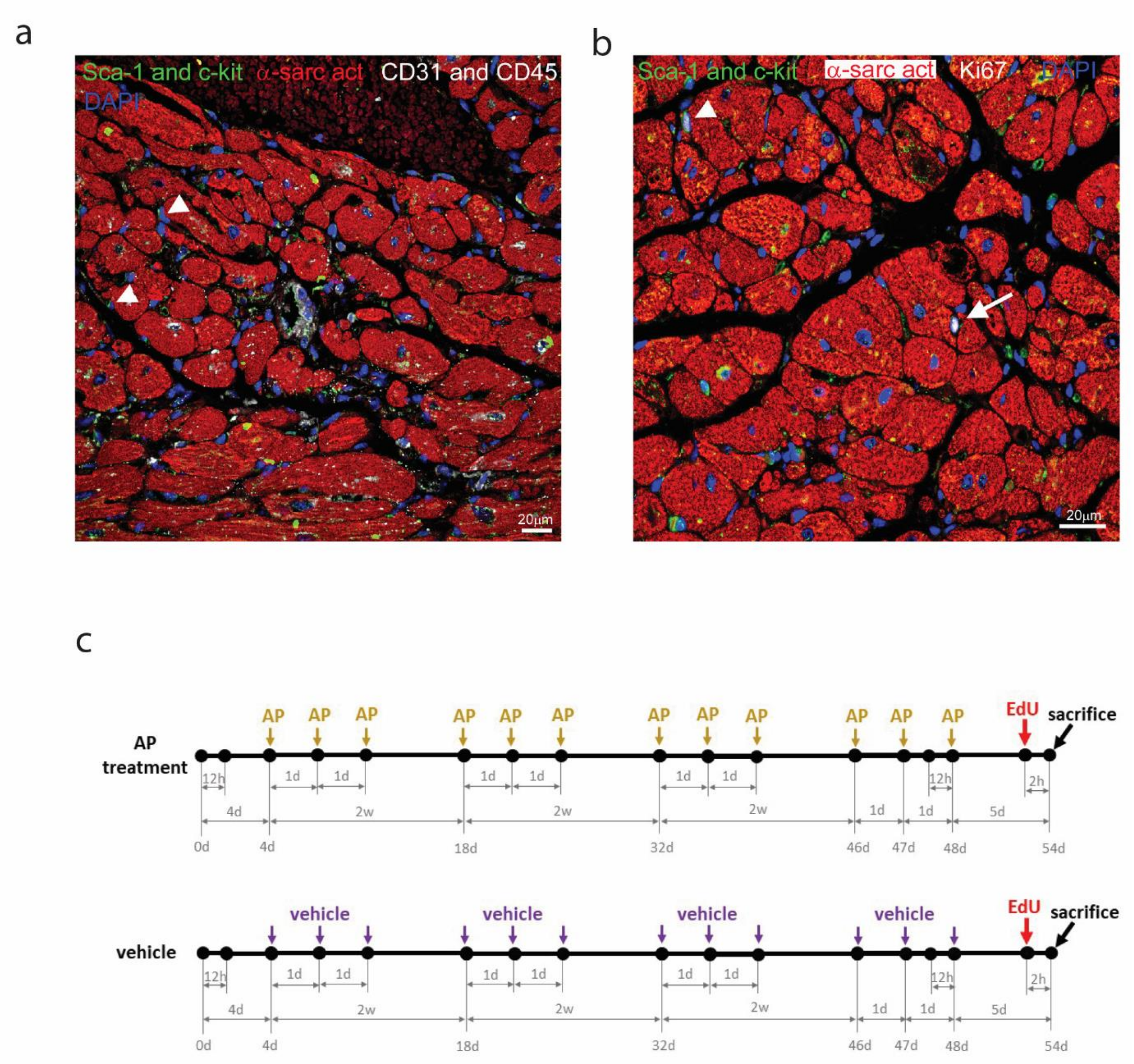

Supplementary Figure 10. Elimination of senescent cells *in vivo* activates resident CPCs (a) Representative confocal image of D+Q-treated INK-ATTAC cardiac cross section showing CPCs (a; arrowheads), which are Sca-1-, c-kit-positive (green) and CD45-, CD31-negative (white). (b) Representative confocal image of AP20187-treated INK-ATTAC cardiac cross section showing a Ki67-positive (white) CPC (arrowhead; green). Arrow indicates a Ki67-positive (white) cell (Sca-1, c-kit-negative). Nuclei are stained blue by DAPI. (c) Experimental design utilised in EdU labelling experiments.

Supplementary Table 1. Conjugated antibodies used for magnetic activated cell sorting (MACS) or flow cytometry (FC) analysis.

	Antigen	Application	Speciation	Isotype	Reactivity	Conjugate	Supplier
**1**	CD117	MACS	Mouse	IgG1	Human	Magnetic	Miltenyi (130-091-332)
**2**	CD45	MACS	Mouse	IgG2a	Human	Magnetic	Miltenyi (130-045-801)
**3**	CD31	MACS	Mouse	IgG1	Human	Magnetic	Miltenyi (130-091-935)
**4**	c-Kit	FC	Mouse	IgG1	Human	PE	Miltenyi (130-098-212)
**5**	CD45	FC	Mouse	IgG1	Human	PE	Biolegend (368510)
**6**	CD31	FC	Mouse	IgG1	Human	PE	Biolegend (303106)
**7**	CD34	FC	Mouse	IgG1	Human	PE	Biolegend (343606)
**8**	CD90	FC	Mouse	IgG1	Human	PE	Biolegend (328110)
**9**	CD166	FC	Mouse	IgG1	Human	PE	Biolegend (34)
**10**	CD105	FC	Mouse	IgG1	Human	PE	Biolegend (323206)
**11**	CD140a	FC	Mouse	IgG1	Human	PE	Biolegend (323506)

Supplementary Table 2. Antibodies used for immunocytochemical (ICC) and immunohistochemical (IHC) analysis.

	Antigen	Application	Speciation	Isotype	Reactivity	Clonality	Supplier
**1**	c-Kit	ICC	Rabbit	IgG1	Human	Polyclonal	DAKO (A4502)
**2**	CD45	ICC	Rabbit	IgG	Human	Polyclonal	Abcam (ab10558)
**3**	CD31	ICC	Rabbit	IgG	Human	Polyclonal	Abcam (ab28364)
**4**	CD34	ICC	Rabbit	IgG	Human	Monoclonal	Abcam (ab81289)
**5**	$p16^{Ink4a}$	ICC	Mouse	IgG2b	Human	Monoclonal	Abcam (ab54210)
**6**	γH2AX	ICC	Rabbit	IgG	Human	Monoclonal	Cell Signalling (#7631)
**7**	Ki67	ICC	Rabbit	IgG	Human	Monoclonal	Vector Labs (VP-K451)
**8**	Nkx2.5	ICC	Goat	IgG	Human	Polyclonal	R&D (AF2444)
**9**	BrdU/EdU	ICC/IHC	Mouse	IgG	-	Monoclonal	Roche (000000011299964001)
**10**	α-Sarc	ICC/IHC	Mouse	IgM	Human/mouse	Monoclonal	Sigma (A2171)
**11**	vWF	ICC/IHC	Rabbit	IgG	Human/Mouse	Polyclonal	DAKO (A0082)
**12**	Sca-1	IHC	Rat	IgG2a	Mouse	Monoclonal	R&D (AF1226)
**13**	c-kit	IHC	Goat	IgG	Mouse	Polyclonal	R&D (AF1356)
**14**	CD45	IHC	Rabbit	IgG	Mouse	Polyclonal	Abcam (ab10558)
**15**	CD34	IHC	Rabbit	IgG	MouseI	Monoclonal	Abcam (ab81289)
**16**	CD31	IHC	Rabbit	IgG	Mouse	Polyclonal	Abcam (ab28364)
**17**	Ki67	IHC	Rabbit	IgG	Mouse	Polyclonal	Abcam (ab16667)

Supplementary Table 3. Primers used in qRT-PCR analysis.

Gene	Forward Primer	Reverse Primer	Tm (°C)	Accession number
GAPDH	GTCAAGGCTGAGAACGGGAA	AAATGAGCCCCAGCCTTCTC	60	NM_002046.6
TBP	AGTGACCCAGCATCACTGTTTC	GCAGGCTGTTGTTCTGATCC	60	NM_003194.4
ß-Actin	GTGGCATCCACGAAACTACC	GTACTTGCGCTCAGGAGGAG	60	NM_001101.3
MMP-3	TGAGGACACCAGCATGAACC	TCAGGGGGAGGTCCATAGAG	60	NM_002422.4
GM-CSF	AATGTTTGACCTCCAGGAGCC	CCCTTGGTCCCTCCAAGATG	60	NM_000758.3
IGFBP-5	GAAAGCAGTGCAAACCTTCCC	AGGTGTGGCACTGAAAGTCC	60	NM_000599.3
IL-8	ACCGGAAGGAACCATCTCAC	GGCAAAACTGCACCTTCACAC	60	NM_000584.3
IL-7	TCCGCTTCCAATAACCCAGC	TGGTTTTCTTCCTTTAACCTGGC	60	NM_000880.3
IL-1ß	ACCAAACCTCTTCGAGGCAC	GCTGCTTCAGACACTTGAGC	60	NM_000576.2
CCL-11	GTCCCCAGAAAGCTGTGATCTTC	GAACATTGCCCACACGTGAC	60	NM_002986.2
CCL-7	GGGAAGCTGTAATCTTCAAGACC	GTCCTGGACCCACTTCTGTG	60	NM_006273.3
CCL-3	GCAGCAGACAGTGGTCAGTC	GCAGCAAGTGATGCAGAGAAC	60	NM_002983.2
CXCL-5	ATCTTCGCTCCTCCAATCTCC	CAGTTTTCCTTGTTTCCACCGTC	60	NM_002994.4
IL-6	TCAATGAGGAGACTTGCCTGG	ATTTGTGGTTGGGTCAGGGG	60	NM_000600.4
CXCL-1	CCGAAGTCATAGCCACACTC	AGCCCCTTTGTTCTAAGCCAG	60	NM_001511.3
PAI-1	CATCCTGGAACTGCCCTACC	AGGGAGAACTTGGGCAGAAC	60	NM_000602.4
CCL-2	TTCTGTGCCTGCTGCTCATAG	TGGGGCATTGATTGCATCTGG	60	NM_002982.3
IGFBP-3	GCGCCAGGAAATGCTAGTGAG	CAACTTTGTAGCGCTGGCTG	60	NM_001013398.1
FGF-2	TGTAGAAGATGTGACGCCGC	TAGCTTGATGTGAGGGTCGC	60	NM_002006.4
TGFß	CTCACCAACCAAAGCCCGAC	TCCACATAGGGCTCAACACG	60	NM_006116.2

References

Baker, D. J., T. Wijshake, T. Tchkonia, N. K. LeBrasseur, B. G. Childs, B. van de Sluis, J. L. Kirkland and J. M. van Deursen (2011). "Clearance of p16Ink4a-positive senescent cells delays ageing-associated disorders." Nature **479**: 232.

Debacq-Chainiaux, F., J. D. Erusalimsky, J. Campisi and O. Toussaint (2009). "Protocols to detect senescence-associated beta-galactosidase (SA-βgal) activity, a biomarker of senescent cells in culture and in vivo." Nature Protocols **4**: 1798.

Ellison, G. M., D. Torella, S. Dellegrottaglie, C. Perez-Martinez, A. Perez de Prado, C. Vicinanza, S. Purushothaman, V. Galuppo, C. Iaconetti, C. D. Waring, A. Smith, M. Torella, C. Cuellas Ramon, J. M. Gonzalo-Orden, V. Agosti, C. Indolfi, M. Galiñanes, F. Fernandez-Vazquez and B. Nadal-Ginard (2011). "Endogenous Cardiac Stem Cell Activation by Insulin-Like Growth Factor-1/Hepatocyte Growth Factor Intracoronary Injection Fosters Survival and Regeneration of the Infarcted Pig Heart." Journal of the American College of Cardiology **58**(9): 977-986.

Lewis, F. C., B. J. Henning, G. Marazzi, D. Sassoon, G. M. Ellison and B. Nadal-Ginard (2014). "Porcine Skeletal Muscle-Derived Multipotent PW1(pos)/Pax7(neg) Interstitial Cells: Isolation, Characterization, and Long-Term Culture." Stem Cells Translational Medicine **3**(6): 702-712.

McIlrath, J., S. D. Bouffler, E. Samper, A. Cuthbert, A. Wojcik, I. Szumiel, P. E. Bryant, A. C. Riches, A. Thompson, M. A. Blasco, R. F. Newbold and P. Slijepcevic (2001). "Telomere Length Abnormalities in Mammalian Radiosensitive Cells." Cancer Research **61**(3): 912.

Roos, C. M., B. Zhang, A. K. Palmer, M. B. Ogrodnik, T. Pirtskhalava, N. M. Thalji, M. Hagler, D. Jurk, L. A. Smith, G. Casaclang-Verzosa, Y. Zhu, M. J. Schafer, T. Tchkonia, J. L. Kirkland and J. D. Miller (2016). "Chronic senolytic treatment alleviates established vasomotor dysfunction in aged or atherosclerotic mice." Aging Cell **15**(5): 973-977.

Smith, A. J., F. C. Lewis, I. Aquila, C. D. Waring, A. Nocera, V. Agosti, B. Nadal-Ginard, D. Torella and G. M. Ellison (2014). "Isolation and Characterization of Resident Endogenous c-Kit+ Cardiac Stem Cells from the Adult Mouse and Rat Heart." Nature Protocols **9**: 1662.

Vicinanza, C., I. Aquila, M. Scalise, F. Cristiano, F. Marino, E. Cianflone, T. Mancuso, P. Marotta, W. Sacco, F. C. Lewis, L. Couch, V. Shone, G. Gritti, A. Torella, A. J. Smith, C. M. N. Terracciano, D. Britti, P. Veltri, C. Indolfi, B. Nadal-Ginard, G. M. Ellison-Hughes and D. Torella (2017). "Adult Cardiac Stem Cells are Multipotent and Robustly Myogenic: C-kit Expression is Necessary but not Sufficient for their Identification." Cell Death And Differentiation **24**: 2101.

Xu, M., A. K. Palmer, H. Ding, M. M. Weivoda, T. Pirtskhalava, T. A. White, A. Sepe, K. O. Johnson, M. B. Stout, N. Giorgadze, M. D. Jensen, N. K. LeBrasseur, T. Tchkonia and J. L. Kirkland (2015). "Targeting senescent cells enhances adipogenesis and metabolic function in old age." eLife **4**: e12997.

Xu, M., T. Tchkonia, H. Ding, M. Ogrodnik, E. R. Lubbers, T. Pirtskhalava, T. A. White, K. O. Johnson, M. B. Stout, V. Mezera, N. Giorgadze, M. D. Jensen, N. K. LeBrasseur and J. L. Kirkland (2015). "JAK inhibition alleviates the cellular senescence-associated secretory phenotype and frailty in old age." Proceedings of the National Academy of Sciences **112**(46): E6301-E6310.
